# Supplementary material for: Lost in translation: how can education about dementia be effectively integrated into medical school contexts? A realist synthesis
Source: BMJ Open. 2023 Nov 17;13(11):e077028. doi: 10.1136/bmjopen-2023-077028 (PMC10660641; doi:10.1136/bmjopen-2023-077028)
Supplement: Supplementary data [file bmjopen-2023-077028supp001.pdf]

BMJ Publishing Group Limited (BMJ) disclaims all liability and responsibility arising from any reliance placed on this supplemental material which has been supplied by the author(s)

Appendix 1. Summary of evolution of research project

Initial title: Systematic Review: improving knowledge, skills and attitudes of undergraduate medical students learning to care for older people with cognitive impairment

Modification of **methodology**: Realist review. Which teaching methods are effective in which context?

Modification of **scope:** Exclusion of delirium which has a more acute presentation, in contrast to dementia which requires a longitudinal understanding of the trajectory of the illness and the changing relationship between PWD and their carers

Modification of **title**: How can we support undergraduate medical students to learn to care for older people with dementia? A realist synthesis

Modification of **purpose:** Move away from what is effective to why effective interactive are not widely implemented within curricula

Modification of **title:** What are the barriers and facilitators to integrating effective UG education about dementia into UG medical curricula?

Finalised title: Lost in translation: How can education about dementia be effectively integrated into medical school contexts? A realist synthesis
