# Supplementary material for: Lost in translation: how can education about dementia be effectively integrated into medical school contexts? A realist synthesis
Source: BMJ Open. 2023 Nov 17;13(11):e077028. doi: 10.1136/bmjopen-2023-077028 (PMC10660641; doi:10.1136/bmjopen-2023-077028)
Supplement: Supplementary data [file bmjopen-2023-077028supp002.pdf]

Supplementary file – full search strategies

PubMed 27/04/21

(((Schools, Medical [MeSH] OR Students, medical [MeSH]) OR Education, medical undergraduate [MeSH]) AND Neurocognitive disorders [MeSH] = 98

Embase 27/04/21[1]

(((Medical student (subject heading) OR undergraduate student (subject heading) OR medical school (subject heading)) AND Cognitive defect (subject heading) = 249

CINAHL 06/10/20[2]

students, medical (CINAHL term) AND delirium, dementia, amnestic, cognitive disorder (CINAHL term) = 0

Psycinfo 27/04/21[3]

(Medical students[4] (subject heading) OR Medical education (subject heading)) AND Cognitive Impairment (subject heading) = 11

No further limitations (eg year of publication or language) applied

---

All databases: 98+249+11 = 358

Eligibility criteria:

Undergraduate or graduate entry medical school programme

Teaching and learning focussing on dementia (rather than delirium or cognitive impairment more broadly)

Evaluating student outcomes > satisfaction, knowledge, skills, attitudes or behaviours

Interventions described clearly enough to classify teaching method

Any research design including quantitative and qualitative

English language

>23 duplicates

>4 foreign language (1 Dutch, 3 German)

>238 excluded by title

= 93 to review in abstract or full text

[1] Search using Ovid Technologies via Newcastle University
[2] Search using CINAHL Information Systems via Newcastle University
[3] Search using American Psychological Association via Newcastle University
[4] Cannot search using term "undergraduate"

Of 93, 77 excluded[5] = 16 eligible papers

[5] Reasons for exclusions could be provided as a itemised list but include: baseline learning needs survey, not specific to dementia education, commentary or clinical intervention.
