# Supplementary material for: Lost in translation: how can education about dementia be effectively integrated into medical school contexts? A realist synthesis
Source: BMJ Open. 2023 Nov 17;13(11):e077028. doi: 10.1136/bmjopen-2023-077028 (PMC10660641; doi:10.1136/bmjopen-2023-077028)
Supplement: Author's manuscript [file bmjopen-2023-077028.draft_revisions.pdf]

BMJ Open is committed to open peer review. As part of this commitment we make the peer review history of every article we publish publicly available.

When an article is published we post the peer reviewers' comments and the authors' responses online. We also post the versions of the paper that were used during peer review. These are the versions that the peer review comments apply to.

The versions of the paper that follow are the versions that were submitted during the peer review process. They are not the versions of record or the final published versions. They should not be cited or distributed as the published version of this manuscript.

BMJ Open is an open access journal and the full, final, typeset and author-corrected version of record of the manuscript is available on our site with no access controls, subscription charges or pay-per-view fees (http://bmjopen.bmj.com).

If you have any questions on BMJ Open's open peer review process please email info.bmjopen@bmj.com

BMJ Open

Lost in translation: How can education about dementia be effectively integrated into medical school contexts? A realist synthesis

Journal:	*BMJ Open*
Manuscript ID	bmjopen-2023-077028
Article Type:	Original research
Date Submitted by the Author:	23-Jun-2023

Complete List of Authors:	Tullo, Ellen; University of Sunderland, Sunderland Medical School<br>Wakeling, Luisa; Newcastle University, School of Dental Sciences<br>Pearse, Rachel; Northumbria Healthcare NHS Foundation Trust<br>Kheng Khoo, Tien; Griffith University, School of Medicine & Dentistry<br>Teodorczuk, Andrew; The University of Queensland, Faculty of Medicine; The Prince Charles Hospital

Keywords:	Dementia, MEDICAL EDUCATION & TRAINING, EDUCATION & TRAINING (see Medical Education & Training)

SCHOLARONE™
Manuscripts

I, the Submitting Author has the right to grant and does grant on behalf of all authors of the Work (as defined in the below author licence), an exclusive licence and/or a non-exclusive licence for contributions from authors who are: i) UK Crown employees; ii) where BMJ has agreed a CC-BY licence shall apply, and/or iii) in accordance with the terms applicable for US Federal Government officers or employees acting as part of their official duties; on a worldwide, perpetual, irrevocable, royalty-free basis to BMJ Publishing Group Ltd ("BMJ") its licensees and where the relevant Journal is co-owned by BMJ to the co-owners of the Journal, to publish the Work in this journal and any other BMJ products and to exploit all rights, as set out in our licence.

The Submitting Author accepts and understands that any supply made under these terms is made by BMJ to the Submitting Author unless you are acting as an employee on behalf of your employer or a postgraduate student of an affiliated institution which is paying any applicable article publishing charge ("APC") for Open Access articles. Where the Submitting Author wishes to make the Work available on an Open Access basis (and intends to pay the relevant APC), the terms of reuse of such Open Access shall be governed by a Creative Commons licence – details of these licences and which Creative Commons licence will apply to this Work are set out in our licence referred to above.

Other than as permitted in any relevant BMJ Author's Self Archiving Policies, I confirm this Work has not been accepted for publication elsewhere, is not being considered for publication elsewhere and does not duplicate material already published. I confirm all authors consent to publication of this Work and authorise the granting of this licence.

For peer review only

Lost in translation: How can education about dementia be effectively integrated into medical school contexts? A realist synthesis

Acknowledgments: with thanks to __ for her advice on the realist approach and structuring our report.

Disclosure of interests: the authors report no conflict of interest.

Funding: the authors report no funding associated with this work.

Data access statement: No new data were created during this study.

Open Access: This is an open access article distributed in accordance with the Creative Commons Attribution Non Commercial (CC BY-NC 4.0) license, which permits others to distribute, remix, adapt, build upon this work non-commercially, and license their derivative works on different terms, provided the original work is properly cited, appropriate credit is given, any changes made indicated, and the use is non-commercial. See: http://creativecommons.org/licenses/by-nc/4.0/.

Contribution summary:

__ developed the idea and approach, constructed the research team and prepared the manuscript. ___ was responsible for the primary search of initial studies. All authors contributed to the search refinement, data extraction, analysis and review of the manuscript. All authors accepted the final version.

For peer review only - http://bmjopen.bmj.com/site/about/guidelines.xhtml

Lost in translation: How can education about dementia be effectively integrated into medical school contexts? A realist synthesis

Objectives

The prevalence of dementia in both community and hospital settings requires a clinical workforce that is skilled in diagnosis and management of the condition to competently care for patients. Though evidence of successful educational interventions about dementia exist, effective translation into medical school curricula is the exception rather than the norm.

Design

We adopted a realist synthesis approach following RAMESES guidelines to answer the following questions: i) what are the barriers to integrating effective interventions about dementia into medical school curricula, ii) where they are successfully delivered, what are the contextual factors that allow for this enactment?

Results

Our initial literature search identified sixteen relevant papers for review. Systematic extraction of data informed the development of an initial programme theory structured around four contextual barriers: “culture”, “concern for patient welfare”, “student attitudes” and “logistics” with associated facilitatory mechanisms embed medical education about dementia.

Conclusions

We outline the process of generating our initial programme theory, including overlap with Cultural Historical Activity Theory (CHAT). We outline our intention to refine our programme theory through ongoing review of the evidence base and collaboration with

For peer review only - http://bmjopen.bmj.com/site/about/guidelines.xhtml

stakeholders, with the aim of finalising a model for successful integration of dementia education.

Key words: cultural historical activity theory, curriculum, dementia, realist synthesis

Strengths and limitations of this study:

- Realist reviews allow flexibility to refine study methodology in parallel with literature review in order to explain how complex educational interventions might work (or not) in particular contexts
- A realist review deciphers what works for whom, in what circumstances, offering a more pragmatic answer for educators interested in improving medical education about dementia in their own institutions than a traditional systematic review
- Our study sets out an initial programme theory that can be used as a tool to help educators integrate effective education about dementia into the undergraduate curriculum internationally
- Our initial programme theory has not yet been reviewed and refined by stakeholders outside of our study team
- The longitudinal viability of integrating dementia education into the undergraduate curriculum remains unanswered by this study

Introduction

With the ageing population, dementia is an increasingly prevalent illness. Prevalence increases with age such that one in six people in the UK aged over 80 have dementia. (1) Moreover, there is evidence internationally that care for patients with dementia is poor, and staff feel unequipped to manage people with dementia (PWD). (2, 3) In part this can be attributed to gaps in skills and knowledge, (4) but also attitudes. (5, 6) Attitudes are shaped by early education experiences and doctors as leaders in healthcare have an important role in setting the compass of care. (7)

Unfortunately, education about dementia in medical schools remains patchy. (8) There is piecemeal evidence from international studies that discrete interventions can improve students' knowledge of and attitudes towards PWD. Even brief interventions that incorporate elements of experiential learning such as simulation; (9-11) and storytelling (12) have been

For peer review only - http://bmjopen.bmj.com/site/about/guidelines.xhtml

shown to have at least a short-term positive impact on student knowledge and attitudes, although longer-term impacts and sustainability remain unclear. Care home placements and clinical clerkships facilitating contact with PWD likewise appear to improve knowledge and attitudes, (13, 14)

but are infrequently implemented.

There are also examples of more extensive educational interventions in the form of longitudinal pairings of medical students with PWD. In the United States (US) the PAIRS Program, (15) developed from an original Buddy Program (16)

brings together medical students and PWD over a period of one year.

Mixed methods evaluation suggests enhanced knowledge, skills and positive attitudes amongst medical students who participated, although students were self-selected and therefore likely to have held predisposing positive attitudes.

The PAIRS model has been adapted in the form of the two-year *Time for Dementia* programme, run collaboratively between a medical school and the UK Alzheimer's Society. (17) Evaluation of *Time for Dementia* is ongoing, but there is evidence from a controlled trial that the intervention led to improvements in medical students' knowledge and attitudes, (18) and also has potential benefits for the patients and families. (19)

Longitudinal programmes involving PWD however are the exception rather than the norm – medical school education about dementia remains inadequate to sufficiently equip graduates to meet the needs of PWD. Given the imperative to improve care for PWD and the existence of evidence as to effective practice, it remains unclear why educational interventions about dementia are not implemented more widely across medical schools. Arguably, this is because curricula are predicated on rigid historically structured content; to bring in change would involve challenging the traditional status quo as "ologies" risk being replaced and thereby devalued.

For peer review only - http://bmjopen.bmj.com/site/about/guidelines.xhtml

Rationale and research intent

Our initial intention was to review existing educational interventions about dementia to identify which features of such interventions led to enhancement in medical students' knowledge, skills, attitudes and behaviours. However, our initial review of the literature, including studies cited above, suggested sufficient evidence of a range of effective models of teaching and learning about dementia. Aligned to the research intent the more pertinent questions are, firstly, why effective interventions are not more frequently and systematically integrated into medical school curricula? Secondly, for whom and in which circumstances is successful dementia education effectively implemented in medical school curricula? We reasoned that this question might be better answered using a realist synthesis rather than a more traditional systematic review. (20) Such an approach aims to decipher what works for whom, in what circumstance and is therefore likely to provide a more helpful answer for educators interested in improving medical education about dementia in their own institutions.

Methods

Changes to the review process

As outlined above, the focus and purpose of our review has already evolved. An accepted feature of realist synthesis is that purpose, scope, inclusion criteria and method of data synthesis are refined in an iterative manner as the review progresses. (21) Details of the evolution of our study purpose is included as Appendix 1. Our report is structured according to RAMESES publication standards. (22)

Justification for realist synthesis

We have suggested that there is already sufficient evidence as to effective methods for delivering medical education about dementia and an implementation gap. Therefore, a traditional systematic review to highlight key features of effective practice is unlikely to

For peer review only - http://bmjopen.bmj.com/site/about/guidelines.xhtml

answer the question as to why education about dementia remains under-represented in medical curricula or align to the research intent. Rather, there is a need to improve understanding of the barriers to implementation and the facilitators in institutions that have successfully introduced effective practice.

Realist reviews add explanation as to how interventions might work (or not) in particular contexts to produce change. (23) The determinant of a desired outcome, in this case improving medical students' ability to care for PWD, are unlikely to be simple or linear – rather, multiple mechanisms are likely to be relevant. (21) These underlying mechanisms may not be intrinsic to the educational intervention itself, but dependent on the historical context, location and the participants involved. For example, in the case of dementia, public and professional attitudes (sometimes ageist) have changed significantly over the last decades, (5) and this may have a bearing on the gap between the expanding needs of PWD and its proportional under-representation in the medical curriculum. A traditional systematic review is unlikely to capture this contextual issue.

A realist approach has been used to examine the complex underpinnings of a range of polemics in medical education including supporting international medical graduates into the UK workforce, (24) the supervision and appraisal process of doctors (25, 26), and how healthcare training translates into benefits for patients. (27) A realist synthesis will similarly allow us to develop a "programme theory" – a diagram to lay out the contexts, mechanisms and outcomes in relation to the curricular integration of UG education about dementia.

Exploratory literature scoping

We drafted the following eligibility criteria before conducting an exploratory search of databases in (PubMed, Embase, CINAHL, Psychinfo) using the MesH terms *Schools, Medical; Students, Medical; Education, Medical* AND *Neurocognitive disorders,* or the closest possible set of terms within each database.

For peer review only - http://bmjopen.bmj.com/site/about/guidelines.xhtml

Eligibility criteria:

- Undergraduate or graduate entry medical school programme
- Teaching and learning focussing on dementia (rather than delirium or cognitive impairment more broadly)
- Evaluating student outcomes > satisfaction, knowledge, skills, attitudes or behaviours
- Interventions described clearly enough to classify teaching method
- Any research design including quantitative and qualitative
- English language

Selection and appraisal of documents

We divided the articles between our research team and constructed a shared spreadsheet to enter key information about eligible studies and to keep track of the reason for excluding studies that did not fit eligibility criteria. We extracted descriptive data about the nature of educational interventions and narrative information as to barriers and facilitators to implementing those interventions. We used the template as a basis for discussion of key findings at regular team meetings and to note any concerns about quality of the included studies.

Analysis and synthesis

We derived our IPT from synthesis of the key barriers and facilitators to implementation emerging from the initial literature review and data extraction as described in the results. This is the first step in our process of realist synthesis – proposed ongoing realist methodology to iteratively test and refine the model is outlined in the discussion.

For peer review only

Results

Literature search

Our initial database searches yielded 358 papers. Twenty-three were duplicates, 238 were excluded by title and four were not in English. We reviewed 93 papers by abstract or full text. Seventy-seven papers did not meet our eligibility criteria, leaving sixteen studies for data extraction (Figure 1).

Manual searching of the reference lists of the sixteen eligible studies, and our team's awareness of pertinent literature beyond the remit of the search highlighted that our initial database search terms had not been sufficiently broad to identify all relevant interventions. This may be due to medical education research being referenced across different database types (biomedical, psychiatric, educational) that each use a range of search terms in relation to dementia, medical education and the pre-qualification stage of medical training. We included a further four relevant studies in our initial synthesis to inform our IPT (Table 1).

Document characteristics

Of the twenty papers evaluating UG dementia education, thirteen involved only medical students and five involved other healthcare professions including pharmacy, physician assistants and nurses. One study involved staff contributing to an educational programme. Cohort sizes ranged from twelve to 386 and the lengths of the educational interventions ranged from one-hour to two-years. The twenty studies considered a range of outcomes including student knowledge (n=7), student attitudes (n=9) and qualitative data collected from focus groups, interviews and student reflections (n=9).

For peer review only - http://bmjopen.bmj.com/site/about/guidelines.xhtml

Initial findings

Extraction of narrative data about barriers to and facilitators of implementation of each intervention revealed a number of common themes. Many studies initially highlighted the barriers towards implementing prior educational interventions about dementia as a rationale for the development of their own.

The evolution of medical education is influenced by the historical and cultural context in which it takes place. There has been a strong reliance on Flexner models of curricula that are over 100 years old - it is surprising how slowly curricular structures and content has evolved. (28) An emphasis on system-based specialties and academic disciplines means that conditions such as dementia do not
'belong' to a particular specialty and therefore may not have systematically appeared in the UG medical curriculum. This has been highlighted by national guidelines in the UK and elsewhere, leading to mandated dementia education, cited by some authors as a prompt for their institution to introduce this. Despite such a mandate, authors note persistent negative attitudes towards dementia amongst faculty which act as a barrier to implementation. The presence of a committed leader and faculty engagement appears to be an important facilitator to integration of dementia education.

Authors cited understandable concerns about the welfare of PWD who might have limited capacity to consent to participation in teaching, and on the potential burden to PWD and their carers. Teams that worked in partnership with PWD, carers, and voluntary sector organisations in the design and delivery of dementia education were able to navigate some of these risks. Other studies elected to use virtual or simulated alternatives rather than direct contact with PWD and their carers.

The attitudes of medical students were also of relevance – there were examples of negative attitudes towards dementia as a topic, with student perception that community-based

For peer review only - http://bmjopen.bmj.com/site/about/guidelines.xhtml

teaching about dementia detracted from the more pertinent environment of acute care. As such, students were less motivated to learn about chronic conditions such as dementia in community placements than they were about acute presentations of illnesses in emergency environments. This may be associated with institutional culture that devalues care of older adults, with the negative attitudes of faculty influencing medical students, sometimes termed the 'hidden curriculum'. Despite negative attitudes, successful programmes of education about dementia instilled motivation in students through mechanisms such as sustained supervision from teachers and peers, and through systematic reflective practice.

Authors also cited the substantial logistical challenges to integration of dementia education, most notably longitudinal programmes pairing medical students with PWD. A full timetable and resource constraints challenged curriculum designers, whereas dedicated resources, administrative support, clear roles for faculty members and iterative implementation acted as facilitators to integrate dementia education.

One paper specifically explored the barriers and facilitators to implementing a longitudinal dementia education programme in a single site. (29) Their findings overlap substantially with the themes appearing across other studies, affording some validity to our initial explanation of contexts, mechanisms and outcomes in the following IPT.

Initial programme theory (IPT):

An IPT (also known as draft or rough programme theory) *'refers to the initial sketch of a theory that is used to guide a realist synthesis'*. (21) We synthesised the findings from our exploratory literature search with our own knowledge and experience of designing and delivering medical education about dementia to produce the following IPT illustrating the contexts where teaching and learning takes place, the mechanisms (barriers and facilitators to integration into the curriculum) and the desired outcomes, often termed CMOs (Figure 2).

For peer review only - http://bmjopen.bmj.com/site/about/guidelines.xhtml

We condensed and structured our initial findings into four contextual barriers to implementation of effective medical education: culture, concern for patient welfare, student attitudes to dementia, and logistics. ‘Culture’ concerns the rigidity of curricular structures and the perceived lower status of conditions such as dementia in the hierarchy of ‘ologies’. ‘Concern for patient welfare’ incorporates legitimate concerns about the potential vulnerability of PWD and their carers to be burdened by involvement in education. ‘Student attitudes’ acknowledges the perception that clinical experience of acute care is a more valuable learning experience than community-based engagement with PWD. ‘Logistics’ highlight the resource implications in terms of time and money to implement longitudinal interventions for large numbers of students. Each of the four contextual barriers are accompanied by mechanisms that facilitate surmounting these barriers in order to achieve the outcome of embedded education which prepares medical students for graduation to practice.

Discussion

Our IPT proposes four categories of barriers to implementation of effective dementia education which derive from the medical school context: culture, concern for patient welfare, student attitudes and logistics. These contextual barriers can be potentially overcome by a range of mechanisms in order to lead to the desired outcome of embedded education that adequately prepares graduates to care for PWD. Realist synthesis encourages the development of programme theory through comparison with existing ‘middle range theories’ – theories available in the wider literature that are relevant to the topic but may come from close or peripheral academic disciplines.

In this case we found that Cultural Historical Activity Theory (CHAT) held some important overlap and relevance with our IPT. CHAT, or social activity theory, is of particular relevance as it is a theory that targets learning at an organisational or systems level and can

For peer review only - http://bmjopen.bmj.com/site/about/guidelines.xhtml

be applied to help bring about educational change. Specifically, it is also relevant in circumstances where multiple voices are present. The fact that the current medical school curricula, and approaches to change, are bound by historical and deeply engrained cultural practice further highlights the relevance as typically it allows challenging of the status quo. It has been applied to dementia settings previously where innovative approaches to systems learning around dementia care have been applied. For example, in the paper by Teodorczuk et al (2015), by defining the object as to learn about the person, its application let to innovative solutions such as relaxing visiting hours to further learn and align with the purpose of the system. (30)

At play are multiple tensions that block embedding of newer dementia-focussed approaches. According to CHAT these can be points of further expansive learning and by this manner a more fit for purpose curriculum can be developed. (31) Current divisions of labour can be identified, and suggestions made to overcome logistical challenges to embedding more dementia focussed curricula. Arguably such an approach will also lead to medical education practice becoming more patient-focussed, when some have critiqued the absence of the patient in medical education. (32)

We propose to use our IPT as the basis for ongoing realist synthesis – we will further interrogate the literature around medical education on dementia to evaluate the fit of our model and make refinements as required. We will test the refined programme theory using case-studies of successful and unsuccessful implementation of educational interventions into medical school curricula. We will seek external validation of the programme theory through consultation with educators with experience of successfully integrating educational interventions about dementia into medical curricula, and those who have faced obstacles to doing so. This will also aid our understanding of the longitudinal viability of integrating dementia education – published articles usually outline the immediate outcomes of an

For peer review only - http://bmjopen.bmj.com/site/about/guidelines.xhtml

intervention, but seldom contain information about the longer-term impact of curriculum reform. Discussions with medical educators who have spent years in the field will help to shed light on a process that can be slow to evolve.

Conclusion

Our IPT provides a firm basis for moving forward with our understanding of effective implementation of dementia teaching into medical education. We acknowledge that the integration of medical education about dementia is likely to be challenging in the context of established medical culture, but the changing demographics of our population and the needs of PWD warrants social accountability to do so. Realist methodology, including the application of systems change models such as CHAT, will help us to better understand the way that contextual barriers to effective medical education about dementia can be overcome.

References

1. Alzheimer's Research UK. *Prevalence.* 2022 Available from: https://www.dementiastatistics.org/statistics-about-dementia/prevalence-2/. [Accessed Sep 2022]

2. Alzheimer's Society. *From diagnosis to end of life: the lived experiences of dementia care and support.* 2020. Available from: https://www.alzheimers.org.uk/sites/default/files/2020-10/pathway_report_full_final.pdf. [Accessed May 2022]

3. [ADI] Alzheimer's Disease International. *World alzheimer report 2021: journey through the diagnosis of dementia*. 2021. Available from: https://www.alzint.org/u/World-Alzheimer-Report-2021.pdf. [Accessed May 2022]

4. Tullo ES, Gordon AL. Teaching and learning about dementia in UK medical schools: a national survey. *BMC Geriatrics*. 2013;13(1):29.

5. [ADI] Alzheimer's Disease International. *World alzheimer report 2019: attitudes to dementia.* 2019 Available from: https://www.alzint.org/u/WorldAlzheimerReport2019.pdf. [Accessed May 2022]

6. Royal College of Psychiatrists. *Who cares wins. Improving the outcome for older people admitted to the general hospital: guidelines for the development of liaison mental health services for older people.* London: Royal College of Psychiatrists (RCPsych); 2005.

For peer review only - http://bmjopen.bmj.com/site/about/guidelines.xhtml

7. Martin J, Dacre J. Professional attitudes: why we should care. *Clin Med (Lond).* 2002;2(3):182-4.

8. Tullo E, Allan L. What should we be teaching medical students about dementia? *Int Psychogeriatr*. 2011;23(7):1044-50.

9. de Abreu ID, Hinojosa-Lindsey M, Asghar-Ali AA. A Simulation Exercise to Raise Learners' Awareness of the Physical and Cognitive Changes in Older Adults. *Acad Psychiatry*. 41(5):684-7.

10. Gilmartin-Thomas JF, McNeil J, Powell A, Malone DT, Larson IC, O'Reilly CL, et al. Qualitative evaluation of how a virtual dementia experience impacts medical and pharmacy students' self-reported knowledge and attitudes towards people with dementia. *Dementia (London).* 2020;19(2):205-20.

11. Matsumura Y, Shinno H, Mori T, Nakamura Y. Simulating Clinical Psychiatry for Medical Students: a Comprehensive Clinic Simulator with Virtual Patients and an Electronic Medical Record System. *Acad Psychiatry.* 2018;42(5):613-21.

12. George DR, Stuckey HL, Whitehead MM. How a creative storytelling intervention can improve medical student attitude towards persons with dementia: a mixed methods study. *Dementia (London)*. 2014;13(3):318-29.

13. Annear MJ, Lea E, Lo A, Tierney L, Robinson A. Encountering aged care: a mixed methods investigation of medical students' clinical placement experiences. *BMC Geriatrics.* 2016;16(1):38.

14. Van Zuilen R, Cabrera K, Danisi J, Palacios J. Achieving medical student mastery in screening for cognitive impairment: results from a blended learning curriculum. *JAGS.* 2018;66 S81-S2.

15. Jefferson AL, Cantwell NG, Byerly LK, Morhardt D. Medical student education program in Alzheimer's disease: The PAIRS Program. *BMC Medical Education*. 2012;12(1):80.

16. Morhardt D. The effects of an experiential learning and mentorship program pairing medical students and persons with cognitive impairment: a qualitative content analysis. [dissertation]. Chicago: Loyola University; 2013.

17. Banerjee S, Farina N, Daley S, Grosvenor W, Hughes L, Hebditch M, et al. How do we enhance undergraduate healthcare education in dementia? A review of the role of innovative approaches and development of the Time for Dementia Programme. *Int J Geriatr Psychiatry.* 2017;32(1):68-75.

18. Banerjee S, Jones C, Wright J, Grosvenor W, Hebditch M, Hughes L, et al. A comparative study of the effect of the Time for Dementia programme on medical students. *Int J Geriatr Psychiatry*. 2021;36(7):1011-9.

19. Cashin Z, Daley S, Hebditch M, Hughes L, Banerjee S. Involving people with dementia and their carers in dementia education for undergraduate healthcare professionals: a qualitative study of motivation to participate and experience. *Int Psychogeriatr.* 2019;31(6):869-76.

For peer review only - http://bmjopen.bmj.com/site/about/guidelines.xhtml

20. Pawson R, Greenhalgh T, Harvey G, Walshe K. Realist review--a new method of systematic review designed for complex policy interventions. *J Health Serv Res Policy*. 2005;10 Suppl 1:21-34.

21. Wong G, Westhorp G, Pawson R, Greenhlagh T. Realist Synthesis. RAMESES Training Materials. 2013. Available from: https://www.ramesesproject.org/media/Realist_reviews_training_materials.pdf. [Accessed July 2022]

22. Wong G, Greenhalgh T, Westhorp G, Buckingham J, Pawson R. RAMESES publication standards: realist syntheses. *BMC Medicine*. 2013;11(1):21.

23. Pawson R, Tilley N. *Realistic Evaluation*. London: Sage; 1997.

24. Kehoe A, McLachlan J, Metcalf J, Forrest S, Carter M, Illing J. Supporting international medical graduates' transition to their host-country: realist synthesis. *Med Educ*. 2016;50(10):1015-32.

25. Brennan N, Bryce M, Pearson M, Wong G, Cooper C, Archer J. Understanding how appraisal of doctors produces its effects: a realist review protocol. *BMJ Open*. 2014;4:e005466.

26. Rees CE, Lee SL, Huang E, Denniston C, Edouard V, Pope K, et al. Supervision training in healthcare: a realist synthesis. *Adv Health Sci Educ Theory Pract.* 2020;25(3):523-61.

27. Illing J, Corbett S, Kehoe A, Carter M, Hesselgreaves H, Crampton P, et al. *How Does the Education and Training of Health and Social Care Staff Transfer to Practice and Benefit Patients? A Realist Approach*. Department of Health; 2019.

28. Jason H, Douglas A. Are the conditions right for a 21st-century medical school? *Lancet*. 2015;385(9969):672-3.

29. Feeney Y, Daley S, Flaherty B, Banerjee S. Barriers and facilitators to implementing a longitudinal dementia education programme into undergraduate healthcare curricula: a qualitative study. *BMC Medical Education*. 2021;21(1):201.

30. Teodorczuk A, Mukaetova-Ladinska E, Corbett S, Welfare M. Deconstructing dementia and delirium hospital practice: using cultural historical activity theory to inform education approaches. *Adv Health Sci Educ Theory Pract*. 2015;20(3):745-64.

31. Teodorczuk A, Morris C. Time to CHAT about globalisation. *Med Educ*. 2019;53(1):3-5.

32. Teodorczuk A, Yardley S, Patel R, Rogers GD, Billett S, Worley P, et al. Medical education research should extend further into clinical practice. *Med Educ*. 2017;51(11):1098-100.

33. Page MJ, McKenzie JE, Bossuyt PM, Boutron I, Hoffmann TC, Mulrow CD, et al. The PRISMA 2020 statement: an updated guideline for reporting systematic reviews. *BMJ*. 2021;327:n71.

For peer review only - http://bmjopen.bmj.com/site/about/guidelines.xhtml

34. Blazek MC, Struble L, Cavalcante A, Masse J. Medical Students in a Dementia Care Facility: an Enhanced Geriatric Psychiatry Experience. *Acad Psychiatry.* 2016;40(6):932-4.

35. George DR, Stuckey HL, Dillon CF, Whitehead MM. Impact of participation in TimeSlips, a creative group-based storytelling program, on medical student attitudes toward persons with dementia: a qualitative study. *Gerontologist*. 2011;51(5):699-703.

36. George DR, Yang C, Stuckey HL, Whitehead MM. Evaluating an arts-based intervention to improve medical student attitudes toward persons with dementia using the dementia attitudes scale. *J Am Geriatr Soc*. 2012;60(8):1583-5.

37. George DR, Stuckey HL, Whitehead MM. An arts-based intervention at a nursing home to improve medical students' attitudes toward persons with dementia. *Acad Med*. 2013;88(6):837-42.

38. Gilmartin-Thomas JF, McNeil J, Powell A, Malone DT, Wolfe R, Larson IC, et al. Impact of a Virtual Dementia Experience on Medical and Pharmacy Students' Knowledge and Attitudes Toward People with Dementia: A Controlled Study. *J Alzheimers Dis.* 2018;62(2):867-76.

39. Goldman JS, Trommer AE. A qualitative study of the impact of a dementia experiential learning project on pre-medical students: a friend for Rachel. *BMC Medical Education*. 2019;19(1):127.

40. Griffiths AW, Cheong WL, Saw PS, Parveen S. Perceptions and attitudes towards dementia among university students in Malaysia. *BMC Medical Education*. 2020;20(1):82.

41. McCaffrey R, Tappen RM, Lichtstein DM, Friedland M. Interprofessional education in community-based Alzheimer's disease diagnosis and treatment. *J Interprof Care*. 2013;27(6):534-6.

42. Yang M, Graham A, Cruz T, Gamaldo C, Salas R, Lehmann S, et al. 3D (dementia, delirium, depression) didactic for medical students *JAGS*. 2016;64:S178.

For peer review only - http://bmjopen.bmj.com/site/about/guidelines.xhtml

Table 1. Papers included in exploratory literature review. (Papers identified through manual searches in **bold).**

Author	Year	Title	Participants	Outcome measure
Annear et al (13)	2016	Encountering aged care: a mixed methods investigation of medical students' clinical placement experiences	61 Y5 MS	Knowledge (DKAT)<br>Qualitative (FG)
**Banerjee et al (17)**	2017	How do we enhance undergraduate healthcare education in dementia?	n/a	n/a
**Banerjee et al (18)**	2021	A comparative study of the effect of the Time for Dementia programme on medical students	386 Y2/3 MS	Knowledge (ADKS, DKQ)<br>Attitudes (ADQ, DAS, MCRS, JSE)
Blazek et al (34)	2015	Medical Students in a Dementia Care Facility: and Enhanced Geriatric Psychiatry Experience	99 Y3 MS	Knowledge (Likert)<br>Attitudes (Likert)<br>Qualitative (FG)
de Abreu et al (9)	2017	A Simulation Exercise to Raise Learners' Awareness of the Physical and Cognitive Changes in Older Adults	49 students (MS, physician assistant, pharmacy, psychiatry residents)	Attitudes (ADQ)
**Feeney at al (29)**	2021	Barriers and facilitators to implementing a longitudinal dementia education programme into undergraduate healthcare curricula: a qualitative study	12 staff	Qualitative (Interviews)
George et al (35)	2011	Impact of participation in TimeSlips, a creative group-based storytelling program, on medical student attitudes toward persons with dementia: a qualitative study.	15 Y4 MS	Qualitative (Questionnaires)
George et al (36)	2012	Evaluating an arts-based intervention to improve medical student attitudes toward persons with dementia using the dementia attitudes scale.	22 Y4 MS	Attitudes (DAS)
George et al (37)	2013	An arts-based intervention at a nursing home to improve medical students' attitudes toward persons with dementia	22 Y4 MS	Attitudes (DAS)
George et al (12)	2014	How a creative storytelling intervention can improve medical student attitude towards persons with dementia: a mixed methods study	22 Y4 MS	Attitudes (DAS)<br>Qualitative (FG)
Gilmartin-Thomas et al (38)	2018	Impact of a Virtual Dementia Experience on Medical and Pharmacy Students' Knowledge and Attitudes Toward People with Dementia: A Controlled Study	278 Y3MS and y4 pharmacy students	Attitudes (DAS)

For peer review only - http://bmjopen.bmj.com/site/about/guidelines.xhtml

**Gilmartin-Thomas et al (10)**	2020	Qualitative evaluation of how a virtual dementia experience impacts medical and pharmacy students' self-reported knowledge and attitudes towards people with dementia	53 Y3 MS and Y4 pharmacy students	Qualitative (FG)
Goldman et al (39)	2019	A qualitative study of the impact of a dementia experiential learning project on pre-medical students: a friend for Rachel	95 pre-MS	Qualitative (reflections)
Griffiths et al (40)	2020	Perceptions and attitudes towards dementia among university students in Malaysia	97 MS and pharmacy students (Y unknown)	Attitudes (AADS, ATOP, IPQ)
Jefferson et al (15)	2012	Medical student education program in Alzheimer's disease: the PAIRS Program.	45 Y1 MS	Knowledge (BPDKT, BUPPDKT)<br>Qualitative (reflections)
Matsumura et al (11)	2018	Simulating Clinical Psychiatry for Medical Students: A Comprehensive Clinic Simulator with Virtual Patients and an Electronic Medical Record System	79 Y5 MS	Knowledge (non-validated)
McCaffrey et al (41)	2013	Interprofessional education in community-based Alzheimer's disease diagnosis and treatment	110 MS and nurse practitioner trainees	Knowledge (non-validated)<br>Attitudes (non-validated)
Morhardt (16)	2013	The effects of an experiential learning and mentorship program pairing medical students and persons with cognitive impairment: A qualitative content analysis.	71 Y1 MS	Qualitative (reflections)
Van Zuilen et al (14)	2018	Achieving medical student mastery in screening for cognitive impairment: Results from a blended learning curriculum	60 MS, (Y unknown)	Knowledge (non-validated)
Yang et al (42)	2016	3D (dementia, delirium, depression) didactic for medical students.	23 Y3/4 MS	Knowledge (non-validated)

AADS – Adolescent Attitude to Dementia Scale
ADKS – Alzheimer's Disease Knowledge Scale
ADQ – Approaches to Dementia Questionnaire
ATOP – Attitudes Towards Older People
BPDKT – Buddy Programme Dementia Knowledge Test
BUPPDKT – Boston University Pairs Programme Knowledge Test
DAS – Dementia Attitudes Scale
DKAT – Dementia Knowledge Assessment Survey
DKQ – Dementia Knowledge Questionnaire
FG – Focus Groups
IPQ – Illness Perception Questionnaire

For peer review only - http://bmjopen.bmj.com/site/about/guidelines.xhtml

JSE – Jefferson Scale of Empathy
MCRS – Medical Condition Regard Scale
MS – medical student
Y - year

For peer review only

For peer review only - http://bmjopen.bmj.com/site/about/guidelines.xhtml

For peer review only

For peer review only - http://bmjopen.bmj.com/site/about/guidelines.xhtml

Figure 1. PRISMA 2020 flow diagram for study selection process (33)

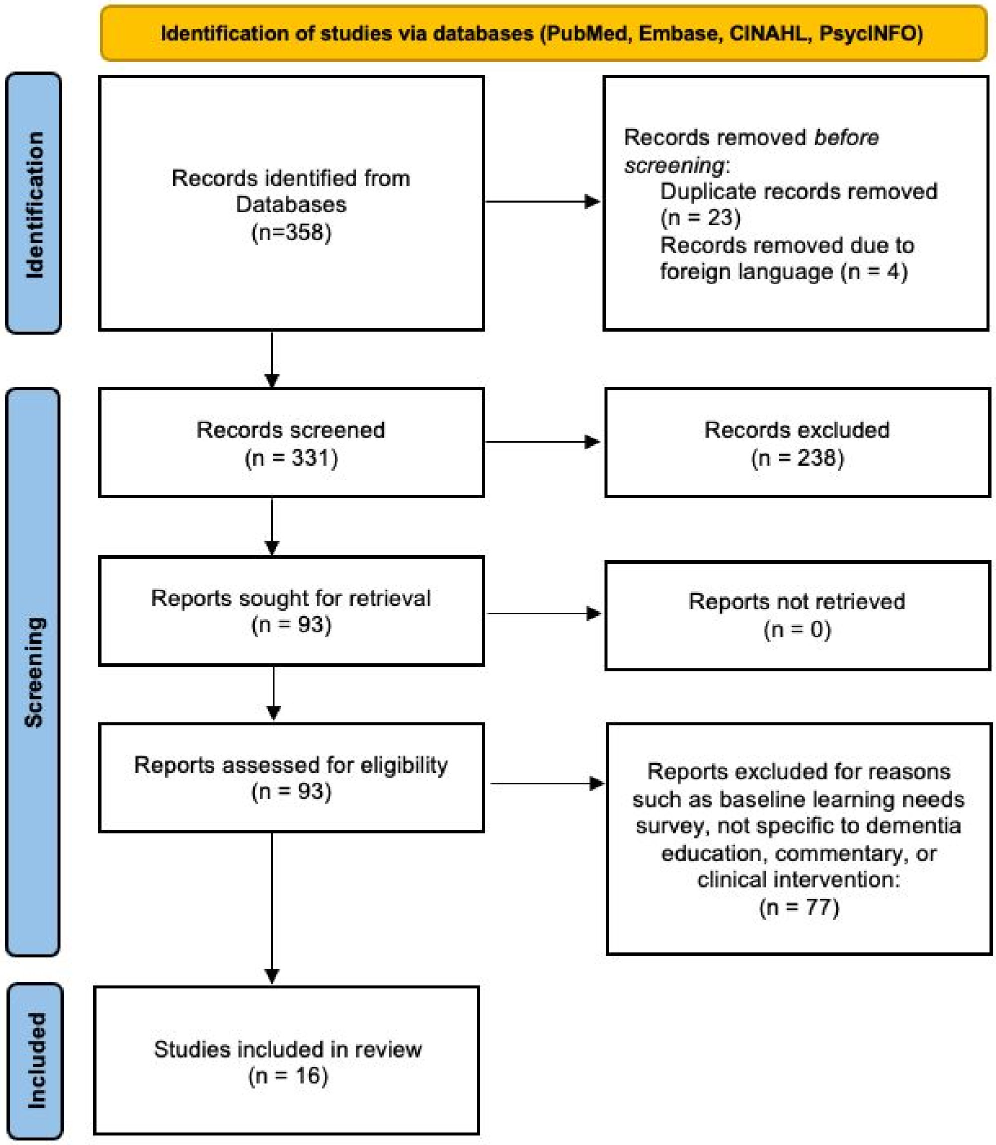

Figure 2. Initial programme theory

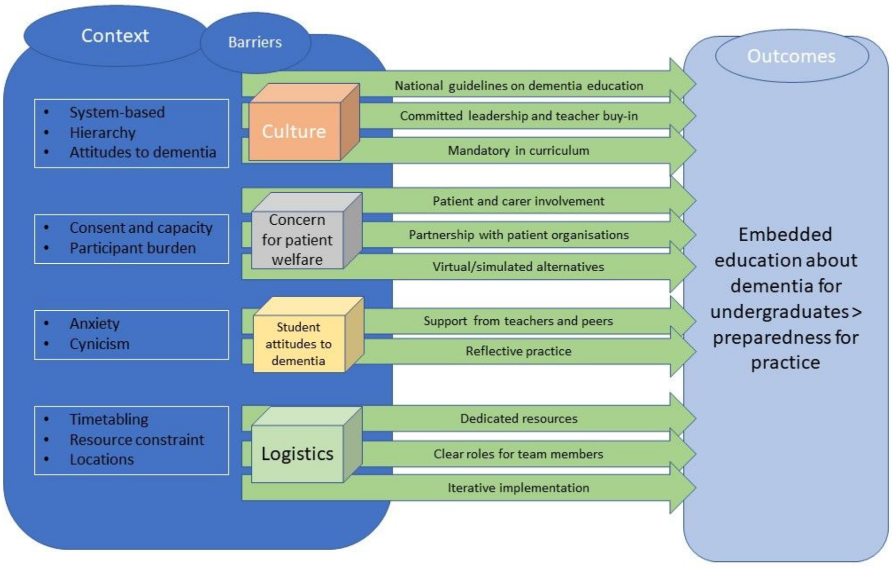

For peer review only

Appendix 1. Summary of evolution of research project

Initial title: Systematic Review: improving knowledge, skills and attitudes of undergraduate medical students learning to care for older people with cognitive impairment

Modification of **methodology**: Realist review. Which teaching methods are effective in which context?

Modification of **scope:** Exclusion of delirium which has a more acute presentation, in contrast to dementia which requires a longitudinal understanding of the trajectory of the illness and the changing relationship between PWD and their carers

Modification of **title**: How can we support undergraduate medical students to learn to care for older people with dementia? A realist synthesis

Modification of **purpose:** Move away from what is effective to why effective interactive are not widely implemented within curricula

Modification of **title:** What are the barriers and facilitators to integrating effective UG education about dementia into UG medical curricula?

Finalised title: Lost in translation: How can education about dementia be effectively integrated into medical school contexts? A realist synthesis

For peer review only

BMJ Open

Lost in translation: How can education about dementia be effectively integrated into medical school contexts? A realist synthesis

Journal:	*BMJ Open*
Manuscript ID	bmjopen-2023-077028.R1
Article Type:	Original research
Date Submitted by the Author:	21-Sep-2023
Complete List of Authors:	Tullo, Ellen; University of Sunderland, Sunderland Medical School<br>Wakeling, Luisa; Newcastle University, School of Dental Sciences<br>Pearse, Rachel; Northumbria Healthcare NHS Foundation Trust<br>Kheng Khoo, Tien; Griffith University, School of Medicine & Dentistry<br>Teodorczuk, Andrew; The University of Queensland, Faculty of Medicine; The Prince Charles Hospital
**Primary Subject Heading**:	Medical education and training
Secondary Subject Heading:	Geriatric medicine
Keywords:	Dementia, MEDICAL EDUCATION & TRAINING, EDUCATION & TRAINING (see Medical Education & Training)

SCHOLARONE™
Manuscripts

I, the Submitting Author has the right to grant and does grant on behalf of all authors of the Work (as defined in the below author licence), an exclusive licence and/or a non-exclusive licence for contributions from authors who are: i) UK Crown employees; ii) where BMJ has agreed a CC-BY licence shall apply, and/or iii) in accordance with the terms applicable for US Federal Government officers or employees acting as part of their official duties; on a worldwide, perpetual, irrevocable, royalty-free basis to BMJ Publishing Group Ltd ("BMJ") its licensees and where the relevant Journal is co-owned by BMJ to the co-owners of the Journal, to publish the Work in this journal and any other BMJ products and to exploit all rights, as set out in our licence.

The Submitting Author accepts and understands that any supply made under these terms is made by BMJ to the Submitting Author unless you are acting as an employee on behalf of your employer or a postgraduate student of an affiliated institution which is paying any applicable article publishing charge ("APC") for Open Access articles. Where the Submitting Author wishes to make the Work available on an Open Access basis (and intends to pay the relevant APC), the terms of reuse of such Open Access shall be governed by a Creative Commons licence – details of these licences and which Creative Commons licence will apply to this Work are set out in our licence referred to above.

Other than as permitted in any relevant BMJ Author's Self Archiving Policies, I confirm this Work has not been accepted for publication elsewhere, is not being considered for publication elsewhere and does not duplicate material already published. I confirm all authors consent to publication of this Work and authorise the granting of this licence.

For peer review only

Lost in translation: How can education about dementia be effectively integrated into medical school contexts? A realist synthesis

Ellen Tullo

Sunderland Medical School, University of Sunderland, UK

Luisa Wakeling (corresponding author)

School of Dental Sciences, Newcastle University, UK

Faculty of Medical Sciences, Newcastle University, Newcastle upon Tyne, NE2 4HH.

luisa.wakeling@ncl.ac.uk

Rachel Pearse

Northumbria NHS Foundation Trust, Newcastle upon Tyne, UK

Tien K Khoo

[3] School of Medicine & Dentistry, Griffith University, Australia

4 Graduate School of Medicine, University of Wollongong, Australia

Andrew Teodorczuk

[1]Faculty of Medicine, The University of Queensland, Australia; [2]The Prince Charles Hospital, Metro North Mental Health, Australia;[3]School of Medicine and Dentistry, Griffith University, Australia

For peer review only

For peer review only - http://bmjopen.bmj.com/site/about/guidelines.xhtml

Lost in translation: How can education about dementia be effectively integrated into medical school contexts? A realist synthesis

Objectives

The prevalence of dementia in both community and hospital settings requires a clinical workforce that is skilled in diagnosis and management of the condition to competently care for patients. Though evidence of successful educational interventions about dementia exist, effective translation into medical school curricula is the exception rather than the norm.

Design

We adopted a realist synthesis approach following RAMESES guidelines to answer the following questions: i) what are the barriers to integrating effective interventions about dementia into medical school curricula, ii) where they are successfully delivered, what are the contextual factors that allow for this enactment?

Data sources

We searched the following databases: PubMed, Embase, CINAHL, Psyc info using the MesH terms *Schools, Medical; Students, Medical; Education, Medical* AND *Neurocognitive disorders,* or the closest possible set of terms within each database.

Eligibility criteria

Undergraduate or graduate entry medical school programme, teaching and learning focussing on dementia, evaluating student outcomes (satisfaction, knowledge, skills, attitudes or behaviours), interventions described clearly enough to classify teaching method, any research design (quantitative and qualitative), English language.

For peer review only - http://bmjopen.bmj.com/site/about/guidelines.xhtml

Data extraction and synthesis

We used a shared spreadsheet to enter key information about eligible studies and the reasons for excluding studies that did not fit eligibility criteria. We extracted descriptive data about the nature of educational interventions and narrative information as to barriers and facilitators to implementing those interventions.

Results

Our initial literature search identified sixteen relevant papers for review. Systematic extraction of data informed the development of an initial programme theory structured around four contextual barriers: “culture”, “concern for patient welfare”, “student attitudes” and “logistics” with associated facilitatory mechanisms embed medical education about dementia.

Conclusions

We outline the process of generating our initial programme theory, including overlap with Cultural Historical Activity Theory (CHAT). We outline our intention to refine our programme theory through ongoing review of the evidence base and collaboration with stakeholders, with the aim of finalising a model for successful integration of dementia education.

Key words: cultural historical activity theory, curriculum, dementia, realist synthesis

Strengths and limitations of this study:

- Realist reviews allow flexibility to refine study methodology in parallel with literature review in order to explain how complex educational interventions might work (or not) in particular contexts.

For peer review only - http://bmjopen.bmj.com/site/about/guidelines.xhtml

For peer review only

- A realist review deciphers what works for whom, in what circumstances, offering a more pragmatic answer for educators interested in improving medical education about dementia in their own institutions than a traditional systematic review.
- Our study sets out an initial programme theory that can be used as a tool to help educators integrate effective education about dementia into the undergraduate curriculum internationally.
- Our initial programme theory has not yet been reviewed and refined by stakeholders outside of our study team.
- The longitudinal viability of integrating dementia education into the undergraduate curriculum remains unanswered by this study.

Introduction

With the ageing population, dementia is an increasingly prevalent illness. Prevalence increases with age such that one in six people in the UK aged over 80 have dementia. (1) Moreover, there is evidence internationally that care for patients with dementia is poor, and staff feel unequipped to manage people with dementia (PWD). (2, 3) In part this can be attributed to gaps in skills and knowledge, (4) but also attitudes. (5, 6) Attitudes are shaped by early education experiences and doctors as leaders in healthcare have an important role in setting the compass of care. (7)

Unfortunately, education about dementia in medical schools remains patchy. (8) There is piecemeal evidence from international studies that discrete interventions can improve students' knowledge of and attitudes towards PWD. Even brief interventions that incorporate elements of experiential learning such as simulation; (9-11) and storytelling (12) have been shown to have at least a short-term positive impact on student knowledge and attitudes, although longer-term impacts and sustainability remain unclear. This may be because of the

For peer review only - http://bmjopen.bmj.com/site/about/guidelines.xhtml

fact that health care settings are not set up to facilitate good dementia practice. Care home placements and clinical clerkships facilitating contact with PWD likewise appear to improve knowledge and attitudes, (13, 14) but are infrequently implemented.

There are also examples of more extensive educational interventions in the form of longitudinal pairings of medical students with PWD. In the United States (US) the PAIRS Program, (15) developed from an original Buddy Program (16) brings together medical students and PWD over a period of one year. Mixed methods evaluation suggests enhanced knowledge, skills and positive attitudes amongst medical students who participated, although students were self-selected and therefore likely to have held predisposing positive attitudes.

The PAIRS model has been adapted in the form of the two-year *Time for Dementia* programme, run collaboratively between a medical school and the UK Alzheimer's Society. (17) Evaluation of *Time for Dementia* is ongoing, but there is evidence from a controlled trial that the intervention led to improvements in medical students' knowledge and attitudes, (18) and also has potential benefits for the patients and families. (19)

Longitudinal programmes involving PWD however are the exception rather than the norm – medical school education about dementia remains inadequate to sufficiently equip graduates to meet the needs of PWD. Given the imperative to improve care for PWD and the existence of evidence as to effective practice, it remains unclear why educational interventions about dementia are not implemented more widely across medical schools. Arguably, this is because curricula are predicated on rigid historically structured content; to bring in change would involve challenging the traditional status quo as "ologies" risk being replaced and thereby devalued. Moreover, systems of care may be slow to adopt good dementia practice and so the hidden curriculum limits the ability to innovate and perceived importance of good dementia education initiatives.

For peer review only - http://bmjopen.bmj.com/site/about/guidelines.xhtml

Rationale and research intent

Our initial intention was to review existing educational interventions about dementia to identify which features of such interventions led to enhancement in medical students' knowledge, skills, attitudes and behaviours. However, our initial review of the literature, including studies cited above, suggested sufficient evidence of a range of effective models of teaching and learning about dementia. Aligned to the research intent the more pertinent questions are, firstly, why effective interventions are not more frequently and systematically integrated into medical school curricula? Secondly, for whom and in which circumstances is successful dementia education effectively implemented in medical school curricula? We reasoned that this question might be better answered using a realist synthesis rather than a more traditional systematic review. (20) Such an approach aims to decipher what works for whom, in what circumstance and is therefore likely to provide a more helpful answer for educators interested in improving medical education about dementia in their own institutions.

Methods

Changes to the review process

As outlined above, the focus and purpose of our review has already evolved. An accepted feature of realist synthesis is that purpose, scope, inclusion criteria and method of data synthesis are refined in an iterative manner as the review progresses. (21) Details of the evolution of our study purpose is included as Appendix 1. Our report is structured according to RAMESES publication standards. (22)

Rationale for realist approach

We have suggested that there is already sufficient evidence as to effective methods for delivering medical education about dementia and an implementation gap. Therefore, a traditional systematic review to highlight key features of effective practice is unlikely to

For peer review only - http://bmjopen.bmj.com/site/about/guidelines.xhtml

answer the question as to why education about dementia remains under-represented in medical curricula or align to the research intent. Rather, there is a need to improve understanding of the barriers to implementation and the facilitators in institutions that have successfully introduced effective practice.

Realist reviews add explanation as to how interventions might work (or not) in particular contexts to produce change. (23) The determinant of a desired outcome, in this case improving medical students' ability to care for PWD, are unlikely to be simple or linear – rather, multiple mechanisms are likely to be relevant. (21) These underlying mechanisms may not be intrinsic to the educational intervention itself, but dependent on the historical context, location and the participants involved. For example, in the case of dementia, public and professional attitudes (sometimes ageist) have changed significantly over the last decades, (5) and this may have a bearing on the gap between the expanding needs of PWD and its proportional under-representation in the medical curriculum. A traditional systematic review is unlikely to capture this contextual issue.

A realist approach has been used to examine the complex underpinnings of a range of polemics in medical education including supporting international medical graduates into the UK workforce, (24) the supervision and appraisal process of doctors (25, 26), and how healthcare training translates into benefits for patients. (27) A realist synthesis will similarly allow us to develop a "programme theory" – a diagram to lay out the contexts, mechanisms and outcomes in relation to the curricular integration of UG education about dementia.

Data sources

We drafted the following eligibility criteria before conducting an exploratory search of databases in (PubMed, Embase, CINAHL, Psychinfo) using the MesH terms *Schools, Medical; Students, Medical; Education, Medical* AND *Neurocognitive disorders,* or the closest possible set of terms within each database (Supplementary file-literature searches).

For peer review only - http://bmjopen.bmj.com/site/about/guidelines.xhtml

Eligibility criteria

- Undergraduate or graduate entry medical school programme
- Teaching and learning focussing on dementia (rather than delirium or cognitive impairment more broadly)
- Evaluating student outcomes > satisfaction, knowledge, skills, attitudes or behaviours
- Interventions described clearly enough to classify teaching method
- Any research design including quantitative and qualitative
- English language

Data extraction and synthesis

We divided the articles between our research team and constructed a shared spreadsheet to enter key information about eligible studies and to keep track of the reason for excluding studies that did not fit eligibility criteria. We extracted descriptive data about the nature of educational interventions and narrative information as to barriers and facilitators to implementing those interventions. We used the template as a basis for discussion of key findings at regular team meetings and to note any concerns about quality of the included studies.

We derived our IPT from synthesis of the key barriers and facilitators to implementation emerging from the initial literature review and data extraction as described in the results. This is the first step in our process of realist synthesis – proposed ongoing realist methodology to iteratively test and refine the model is outlined in the discussion.

Patient and public involvement

None at this stage

For peer review only - http://bmjopen.bmj.com/site/about/guidelines.xhtml

Results

Literature search

Our initial database searches yielded 358 papers. Twenty-three were duplicates, 238 were excluded by title and four were not in English. We reviewed 93 papers by abstract or full text. Seventy-seven papers did not meet our eligibility criteria, leaving sixteen studies for data extraction (Figure 1).

Manual searching of the reference lists of the sixteen eligible studies, and our team's awareness of pertinent literature beyond the remit of the search highlighted that our initial database search terms had not been sufficiently broad to identify all relevant interventions. This may be due to medical education research being referenced across different database types (biomedical, psychiatric, educational) that each use a range of search terms in relation to dementia, medical education and the pre-qualification stage of medical training. We included a further four relevant studies in our initial synthesis to inform our IPT (Table 1).

Document characteristics

Of the twenty papers evaluating UG dementia education, thirteen involved only medical students and five involved other healthcare professions including pharmacy, physician assistants and nurses. One study involved staff contributing to an educational programme. Cohort sizes ranged from twelve to 386 and the lengths of the educational interventions ranged from one-hour to two-years. The twenty studies considered a range of outcomes including student knowledge (n=7), student attitudes (n=9) and qualitative data collected from focus groups, interviews and student reflections (n=9).

For peer review only - http://bmjopen.bmj.com/site/about/guidelines.xhtml

Initial findings

Extraction of narrative data about barriers to and facilitators of implementation of each intervention revealed a number of common themes. Many studies initially highlighted the barriers towards implementing prior educational interventions about dementia as a rationale for the development of their own.

The evolution of medical education is influenced by the historical and cultural context in which it takes place. There has been a strong reliance on Flexner models of curricula that are over 100 years old - it is surprising how slowly curricular structures and content has evolved. (28) An emphasis on system-based specialties and academic disciplines means that conditions such as dementia do not 'belong' to a particular specialty and therefore may not have systematically appeared in the UG medical curriculum. This has been highlighted by national guidelines in the UK and elsewhere, leading to mandated dementia education, cited by some authors as a prompt for their institution to introduce this. Despite such a mandate, authors note persistent negative attitudes towards dementia amongst faculty which act as a barrier to implementation. The presence of a committed leader and faculty engagement appears to be an important facilitator to integration of dementia education.

Authors cited understandable concerns about the welfare of PWD who might have limited capacity to consent to participation in teaching, and on the potential burden to PWD and their carers. Teams that worked in partnership with PWD, carers, and voluntary sector organisations in the design and delivery of dementia education were able to navigate some of these risks. Other studies elected to use virtual or simulated alternatives rather than direct contact with PWD and their carers.

The attitudes of medical students were also of relevance – there were examples of negative attitudes towards dementia as a topic, with student perception that community-based

For peer review only - http://bmjopen.bmj.com/site/about/guidelines.xhtml

teaching about dementia detracted from the more pertinent environment of acute care. As such, students were less motivated to learn about chronic conditions such as dementia in community placements than they were about acute presentations of illnesses in emergency environments. This may be associated with institutional culture that devalues care of older adults, with the negative attitudes of faculty influencing medical students, sometimes termed the 'hidden curriculum'. Despite negative attitudes, successful programmes of education about dementia instilled motivation in students through mechanisms such as sustained supervision from teachers and peers, and through systematic reflective practice.

Authors also cited the substantial logistical challenges to integration of dementia education, most notably longitudinal programmes pairing medical students with PWD. A full timetable and resource constraints challenged curriculum designers, whereas dedicated resources, administrative support, clear roles for faculty members and iterative implementation acted as facilitators to integrate dementia education.

One paper specifically explored the barriers and facilitators to implementing a longitudinal dementia education programme in a single site. (29) Their findings overlap substantially with the themes appearing across other studies, affording some validity to our initial explanation of contexts, mechanisms and outcomes in the following IPT.

Initial programme theory (IPT):

An IPT (also known as draft or rough programme theory) *'refers to the initial sketch of a theory that is used to guide a realist synthesis'*. (21) We synthesised the findings from our exploratory literature search with our own knowledge and experience of designing and delivering medical education about dementia to produce the following IPT illustrating the contexts where teaching and learning takes place, the mechanisms (barriers and facilitators to integration into the curriculum) and the desired outcomes, often termed CMOs (Figure 2).

For peer review only - http://bmjopen.bmj.com/site/about/guidelines.xhtml

We condensed and structured our initial findings into four contextual barriers to implementation of effective medical education: culture, concern for patient welfare, student attitudes to dementia, and logistics. 'Culture' concerns the rigidity of curricular structures and the perceived lower status of conditions such as dementia in the hierarchy of 'ologies'. 'Concern for patient welfare' incorporates legitimate concerns about the potential vulnerability of PWD and their carers to be burdened by involvement in education. 'Student attitudes' acknowledges the perception that clinical experience of acute care is a more valuable learning experience than community-based engagement with PWD. 'Logistics' highlight the resource implications in terms of time and money to implement longitudinal interventions for large numbers of students. Each of the four contextual barriers are accompanied by mechanisms that facilitate surmounting these barriers in order to achieve the outcome of embedded education which prepares medical students for graduation to practice.

Discussion

Our IPT proposes four categories of barriers to implementation of effective dementia education which derive from the medical school context: culture, concern for patient welfare, student attitudes and logistics. These contextual barriers might be overcome by a range of mechanisms in order to lead to the desired outcome of embedded education that adequately prepares graduates to care for PWD. Realist synthesis encourages the development of programme theory through comparison with existing 'middle range theories' – theories available in the wider literature that are relevant to the topic but may come from close or peripheral academic disciplines.

In this case we found that Cultural Historical Activity Theory (CHAT) held important overlap and relevance with our IPT. CHAT, or social activity theory, is of particular relevance as it is a theory that targets learning at an organisational or systems level and can be applied to help

For peer review only - http://bmjopen.bmj.com/site/about/guidelines.xhtml

bring about educational change. Specifically, it is also relevant in circumstances where multiple voices are present. The fact that the current medical school curricula, and approaches to change, are bound by historical and deeply engrained cultural practice further highlights the relevance as typically it allows challenging of the status quo. It has been applied to dementia settings previously where innovative approaches to systems learning around dementia care have been applied. For example, in the paper by Teodorczuk et al (2015), by defining the object as to learn about the person, its application let to innovative solutions such as relaxing visiting hours to further learn and align with the purpose of the system. (30) . Moreover, multiple voices will allow engagement with the practice context and degree of dementia capability that may invariably limit implementation of any education innovations.

At play are multiple tensions that block embedding of newer dementia-focussed approaches. According to CHAT these can be points of further expansive learning and by this manner a more fit for purpose curriculum can be developed. (31) Current divisions of labour both within education and clinical practice can be identified, and suggestions made to overcome logistical challenges to embedding more dementia focussed curricula. Arguably such an approach will also lead to medical education practice becoming more patient-focussed when some have critiqued the absence of the patient in medical education. (32)

A key strength of this realist synthesis has been the flexibility to refine our study methodology in order to explain how complex educational interventions might work (or not) in particular contexts. We believe that this approach will lead to a more pragmatic answer for educators interested in improving medical education about dementia in their own institutions than a traditional systematic review. At this stage our initial programme theory has not yet been reviewed and refined by stakeholders outside of our study team – this limitation will be addressed in the next stage of our project. We will also address the longitudinal viability of

For peer review only - http://bmjopen.bmj.com/site/about/guidelines.xhtml

integrating dementia education into the undergraduate curriculum which remains unanswered at this stage.

We propose to use our IPT as the basis for ongoing realist synthesis – we will further interrogate the literature around medical education on dementia to evaluate the fit of our model and make refinements as required. We will test the refined programme theory using case-studies of successful and unsuccessful implementation of educational interventions into medical school curricula, including neglected areas of teaching that may face similar factors such as learning disability. We will seek external validation of the programme theory through consultation with educators with experience of successfully integrating educational interventions about dementia into medical curricula, in addition to people with dementia and their carers. This will also aid our understanding of the longitudinal viability of integrating dementia education – published articles usually outline the immediate outcomes of an intervention, but seldom contain information about the longer-term impact of curriculum reform. Discussions with medical educators who have spent years in the field will help to shed light on a process that can be slow to evolve.

Conclusion

Our IPT provides a firm basis for moving forward with our understanding of effective implementation of dementia teaching into medical education. We acknowledge that the integration of medical education about dementia is likely to be challenging in the context of established medical culture, but the changing demographics of our population and the needs of PWD warrants social accountability to do so. Realist methodology, including the application of systems change models such as CHAT, will help us to better understand the way that contextual barriers to effective medical education about dementia can be overcome.

For peer review only - http://bmjopen.bmj.com/site/about/guidelines.xhtml

Acknowledgments: with thanks to Millie Kehoe for her advice on the realist approach and structuring our report.

Disclosure of interests: the authors report no conflict of interest.

Funding: the authors report no funding associated with this work.

Data access statement: No new data were created during this study.

Open Access: This is an open access article distributed in accordance with the Creative Commons Attribution Non Commercial (CC BY-NC 4.0) license, which permits others to distribute, remix, adapt, build upon this work non-commercially, and license their derivative works on different terms, provided the original work is properly cited, appropriate credit is given, any changes made indicated, and the use is non-commercial. See: http://creativecommons.org/licenses/by-nc/4.0/.

Contribution summary:

ET conceptualised the idea, approach, and constructed the research team. ET, LW, RP, TK and AT provided feedback and further developed the idea and realist synthesis approach. RP was responsible for the primary search of initial studies. ET, LW, RP, TK and AT contributed to the search refinement, data extraction and analysis. ET summarised findings and drafted an initial programme theory to which LW, RP, TK and AT provided feedback and further developed. ET prepared the initial draft manuscript and LW, RP, TK and AT reviewed and developed the manuscript. All authors (ET, LW, RP, TK and AT) accepted the final version.

Ethics approval:

For peer review only - http://bmjopen.bmj.com/site/about/guidelines.xhtml

Not required for literature review

For peer review only

For peer review only - http://bmjopen.bmj.com/site/about/guidelines.xhtml

Table 1. Papers included in exploratory literature review. (Papers identified through manual searches in **bold).**

Author	Year	Title	Participants	Outcome measure
Annear et al (13)	2016	Encountering aged care: a mixed methods investigation of medical students' clinical placement experiences	61 Y5 MS	Knowledge (DKAT)<br>Qualitative (FG)
**Banerjee et al (17)**	2017	How do we enhance undergraduate healthcare education in dementia?	n/a	n/a
**Banerjee et al (18)**	2021	A comparative study of the effect of the Time for Dementia programme on medical students	386 Y2/3 MS	Knowledge (ADKS, DKQ)<br>Attitudes (ADQ, DAS, MCRS, JSE)
Blazek et al (33)	2015	Medical Students in a Dementia Care Facility: and Enhanced Geriatric Psychiatry Experience	99 Y3 MS	Knowledge (Likert)<br>Attitudes (Likert)<br>Qualitative (FG)
de Abreu et al (9)	2017	A Simulation Exercise to Raise Learners' Awareness of the Physical and Cognitive Changes in Older Adults	49 students (MS, physician assistant, pharmacy, psychiatry residents)	Attitudes (ADQ)
**Feeney at al (29)**	2021	Barriers and facilitators to implementing a longitudinal dementia education programme into undergraduate healthcare curricula: a qualitative study	12 staff	Qualitative (Interviews)
George et al (34)	2011	Impact of participation in TimeSlips, a creative group-based storytelling program, on medical student attitudes toward persons with dementia: a qualitative study.	15 Y4 MS	Qualitative (Questionnaires)
George et al (35)	2012	Evaluating an arts-based intervention to improve medical student attitudes toward persons with dementia using the dementia attitudes scale.	22 Y4 MS	Attitudes (DAS)
George et al (36)	2013	An arts-based intervention at a nursing home to improve medical students' attitudes toward persons with dementia	22 Y4 MS	Attitudes (DAS)
George et al (12)	2014	How a creative storytelling intervention can improve medical student attitude towards persons with dementia: a mixed methods study	22 Y4 MS	Attitudes (DAS)<br>Qualitative (FG)
Gilmartin-Thomas et al (37)	2018	Impact of a Virtual Dementia Experience on Medical and Pharmacy Students' Knowledge and Attitudes Toward People with Dementia: A Controlled Study	278 Y3MS and y4 pharmacy students	Attitudes (DAS)

For peer review only - http://bmjopen.bmj.com/site/about/guidelines.xhtml

**Gilmartin-Thomas et al (10)**	2020	Qualitative evaluation of how a virtual dementia experience impacts medical and pharmacy students' self-reported knowledge and attitudes towards people with dementia	53 Y3 MS and Y4 pharmacy students	Qualitative (FG)
Goldman et al (38)	2019	A qualitative study of the impact of a dementia experiential learning project on pre-medical students: a friend for Rachel	95 pre-MS	Qualitative (reflections)
Griffiths et al (39)	2020	Perceptions and attitudes towards dementia among university students in Malaysia	97 MS and pharmacy students (Y unknown)	Attitudes (AADS, ATOP, IPQ)
Jefferson et al (15)	2012	Medical student education program in Alzheimer's disease: the PAIRS Program.	45 Y1 MS	Knowledge (BPDKT, BUPPDKT)<br>Qualitative (reflections)
Matsumura et al (11)	2018	Simulating Clinical Psychiatry for Medical Students: A Comprehensive Clinic Simulator with Virtual Patients and an Electronic Medical Record System	79 Y5 MS	Knowledge (non-validated)
McCaffrey et al (40)	2013	Interprofessional education in community-based Alzheimer's disease diagnosis and treatment	110 MS and nurse practitioner trainees	Knowledge (non-validated)<br>Attitudes (non-validated)
Morhardt (16)	2013	The effects of an experiential learning and mentorship program pairing medical students and persons with cognitive impairment: A qualitative content analysis.	71 Y1 MS	Qualitative (reflections)
Van Zuilen et al (14)	2018	Achieving medical student mastery in screening for cognitive impairment: Results from a blended learning curriculum	60 MS, (Y unknown)	Knowledge (non-validated)
Yang et al (41)	2016	3D (dementia, delirium, depression) didactic for medical students.	23 Y3/4 MS	Knowledge (non-validated)

AADS – Adolescent Attitude to Dementia Scale
ADKS – Alzheimer's Disease Knowledge Scale
ADQ – Approaches to Dementia Questionnaire
ATOP – Attitudes Towards Older People
BPDKT – Buddy Programme Dementia Knowledge Test
BUPPDKT – Boston University Pairs Programme Knowledge Test
DAS – Dementia Attitudes Scale
DKAT – Dementia Knowledge Assessment Survey
DKQ – Dementia Knowledge Questionnaire
FG – Focus Groups
IPQ – Illness Perception Questionnaire

For peer review only - http://bmjopen.bmj.com/site/about/guidelines.xhtml

JSE – Jefferson Scale of Empathy
MCRS – Medical Condition Regard Scale
MS – medical student
Y - year

For peer review only

Figure 1. PRISMA 2020 flow diagram for study selection process (42)

Figure 2. Initial programme theory

For peer review only - http://bmjopen.bmj.com/site/about/guidelines.xhtml

References

1. Alzheimer's Research UK. *Prevalence.* 2022 Available from: https://www.dementiastatistics.org/statistics-about-dementia/prevalence-2/. [Accessed Sep 2022]

2. Alzheimer's Society. *From diagnosis to end of life: the lived experiences of dementia care and support.* 2020. Available from: https://www.alzheimers.org.uk/sites/default/files/2020-10/pathway_report_full_final.pdf. [Accessed May 2022]

3. [ADI] Alzheimer's Disease International. *World alzheimer report 2021: journey through the diagnosis of dementia*. 2021. Available from: https://www.alzint.org/u/World-Alzheimer-Report-2021.pdf. [Accessed May 2022]

4. Tullo ES, Gordon AL. Teaching and learning about dementia in UK medical schools: a national survey. *BMC Geriatrics*. 2013;13(1):29.

5. [ADI] Alzheimer's Disease International. *World alzheimer report 2019: attitudes to dementia.* 2019 Available from: https://www.alzint.org/u/WorldAlzheimerReport2019.pdf. [Accessed May 2022]

6. Royal College of Psychiatrists. *Who cares wins. Improving the outcome for older people admitted to the general hospital: guidelines for the development of liaison mental health services for older people.* London: Royal College of Psychiatrists (RCPsych); 2005.

7. Martin J, Dacre J. Professional attitudes: why we should care. *Clin Med (Lond).* 2002;2(3):182-4.

8. Tullo E, Allan L. What should we be teaching medical students about dementia? *Int Psychogeriatr*. 2011;23(7):1044-50.

9. de Abreu ID, Hinojosa-Lindsey M, Asghar-Ali AA. A Simulation Exercise to Raise Learners' Awareness of the Physical and Cognitive Changes in Older Adults. *Acad Psychiatry*. 2017;41(5):684-7.

10. Gilmartin-Thomas JF, McNeil J, Powell A, Malone DT, Larson IC, O'Reilly CL, et al. Qualitative evaluation of how a virtual dementia experience impacts medical and pharmacy students' self-reported knowledge and attitudes towards people with dementia. *Dementia (London).* 2020;19(2):205-20.

11. Matsumura Y, Shinno H, Mori T, Nakamura Y. Simulating Clinical Psychiatry for Medical Students: a Comprehensive Clinic Simulator with Virtual Patients and an Electronic Medical Record System. *Acad Psychiatry.* 2018;42(5):613-21.

12. George DR, Stuckey HL, Whitehead MM. How a creative storytelling intervention can improve medical student attitude towards persons with dementia: a mixed methods study. *Dementia (London)*. 2014;13(3):318-29.

13. Annear MJ, Lea E, Lo A, Tierney L, Robinson A. Encountering aged care: a mixed methods investigation of medical students' clinical placement experiences. *BMC Geriatrics.* 2016;16(1):38.

For peer review only - http://bmjopen.bmj.com/site/about/guidelines.xhtml

14. Van Zuilen R, Cabrera K, Danisi J, Palacios J. Achieving medical student mastery in screening for cognitive impairment: results from a blended learning curriculum. *JAGS*. 2018;66 S81-S2.

15. Jefferson AL, Cantwell NG, Byerly LK, Morhardt D. Medical student education program in Alzheimer's disease: The PAIRS Program. *BMC Medical Education*. 2012;12(1):80.

16. Morhardt D. The effects of an experiential learning and mentorship program pairing medical students and persons with cognitive impairment: a qualitative content analysis. [dissertation]. Chicago: Loyola University; 2013.

17. Banerjee S, Farina N, Daley S, Grosvenor W, Hughes L, Hebditch M, et al. How do we enhance undergraduate healthcare education in dementia? A review of the role of innovative approaches and development of the Time for Dementia Programme. *Int J Geriatr Psychiatry*. 2017;32(1):68-75.

18. Banerjee S, Jones C, Wright J, Grosvenor W, Hebditch M, Hughes L, et al. A comparative study of the effect of the Time for Dementia programme on medical students. *Int J Geriatr Psychiatry*. 2021;36(7):1011-9.

19. Cashin Z, Daley S, Hebditch M, Hughes L, Banerjee S. Involving people with dementia and their carers in dementia education for undergraduate healthcare professionals: a qualitative study of motivation to participate and experience. *Int Psychogeriatr*. 2019;31(6):869-76.

20. Pawson R, Greenhalgh T, Harvey G, Walshe K. Realist review--a new method of systematic review designed for complex policy interventions. *J Health Serv Res Policy*. 2005;10 Suppl 1:21-34.

21. Wong G, Westhorp G, Pawson R, Greenhlagh T. Realist Synthesis. RAMESES Training Materials. 2013. Available from: https://www.ramesesproject.org/media/Realist_reviews_training_materials.pdf. [Accessed July 2022]

22. Wong G, Greenhalgh T, Westhorp G, Buckingham J, Pawson R. RAMESES publication standards: realist syntheses. *BMC Medicine*. 2013;11(1):21.

23. Pawson R, Tilley N. *Realistic Evaluation*. London: Sage; 1997.

24. Kehoe A, McLachlan J, Metcalf J, Forrest S, Carter M, Illing J. Supporting international medical graduates' transition to their host-country: realist synthesis. *Med Educ*. 2016;50(10):1015-32.

25. Brennan N, Bryce M, Pearson M, Wong G, Cooper C, Archer J. Understanding how appraisal of doctors produces its effects: a realist review protocol. *BMJ Open*. 2014;4:e005466.

26. Rees CE, Lee SL, Huang E, Denniston C, Edouard V, Pope K, et al. Supervision training in healthcare: a realist synthesis. *Adv Health Sci Educ Theory Pract*. 2020;25(3):523-61.

For peer review only - http://bmjopen.bmj.com/site/about/guidelines.xhtml

27. Illing J, Corbett S, Kehoe A, Carter M, Hesselgreaves H, Crampton P, et al. *How Does the Education and Training of Health and Social Care Staff Transfer to Practice and Benefit Patients? A Realist Approach*. Department of Health; 2019.

28. Jason H, Douglas A. Are the conditions right for a 21st-century medical school? *Lancet*. 2015;385(9969):672-3.

29. Feeney Y, Daley S, Flaherty B, Banerjee S. Barriers and facilitators to implementing a longitudinal dementia education programme into undergraduate healthcare curricula: a qualitative study. *BMC Medical Education*. 2021;21(1):201.

30. Teodorczuk A, Mukaetova-Ladinska E, Corbett S, Welfare M. Deconstructing dementia and delirium hospital practice: using cultural historical activity theory to inform education approaches. *Adv Health Sci Educ Theory Pract*. 2015;20(3):745-64.

31. Teodorczuk A, Morris C. Time to CHAT about globalisation. *Med Educ*. 2019;53(1):3-5.

32. Teodorczuk A, Yardley S, Patel R, Rogers GD, Billett S, Worley P, et al. Medical education research should extend further into clinical practice. *Med Educ*. 2017;51(11):1098-100.

33. Blazek MC, Struble L, Cavalcante A, Masse J. Medical Students in a Dementia Care Facility: an Enhanced Geriatric Psychiatry Experience. *Acad Psychiatry*. 2016;40(6):932-4.

34. George DR, Stuckey HL, Dillon CF, Whitehead MM. Impact of participation in TimeSlips, a creative group-based storytelling program, on medical student attitudes toward persons with dementia: a qualitative study. *Gerontologist*. 2011;51(5):699-703.

35. George DR, Yang C, Stuckey HL, Whitehead MM. Evaluating an arts-based intervention to improve medical student attitudes toward persons with dementia using the dementia attitudes scale. *J Am Geriatr Soc*. 2012;60(8):1583-5.

36 George DR, Stuckey HL, Whitehead MM. An arts-based intervention at a nursing home to improve medical students' attitudes toward persons with dementia. *Acad Med*. 2013;88(6):837-42.

37 Gilmartin-Thomas JF, McNeil J, Powell A, Malone DT, Wolfe R, Larson IC, et al. Impact of a Virtual Dementia Experience on Medical and Pharmacy Students' Knowledge and Attitudes Toward People with Dementia: A Controlled Study. *J Alzheimers Dis*. 2018;62(2):867-76.

38 Goldman JS, Trommer AE. A qualitative study of the impact of a dementia experiential learning project on pre-medical students: a friend for Rachel. *BMC Medical Education*. 2019;19(1):127.

39 Griffiths AW, Cheong WL, Saw PS, Parveen S. Perceptions and attitudes towards dementia among university students in Malaysia. *BMC Medical Education*. 2020;20(1):82.

For peer review only - http://bmjopen.bmj.com/site/about/guidelines.xhtml

40. McCaffrey R, Tappen RM, Lichtstein DM, Friedland M. Interprofessional education in community-based Alzheimer's disease diagnosis and treatment. *J Interprof Care*. 2013;27(6):534-6.

41. Yang M, Graham A, Cruz T, Gamaldo C, Salas R, Lehmann S, et al. 3D (dementia, delirium, depression) didactic for medical students *JAGS*. 2016;64:S178.

42. Page MJ, McKenzie JE, Bossuyt PM, Boutron I, Hoffmann TC, Mulrow CD, et al. The PRISMA 2020 statement: an updated guideline for reporting systematic reviews. *BMJ*. 2021;327:n71

For peer review only

For peer review only

For peer review only - http://bmjopen.bmj.com/site/about/guidelines.xhtml

Figure 1. PRISMA 2020 flow diagram for study selection process (42)

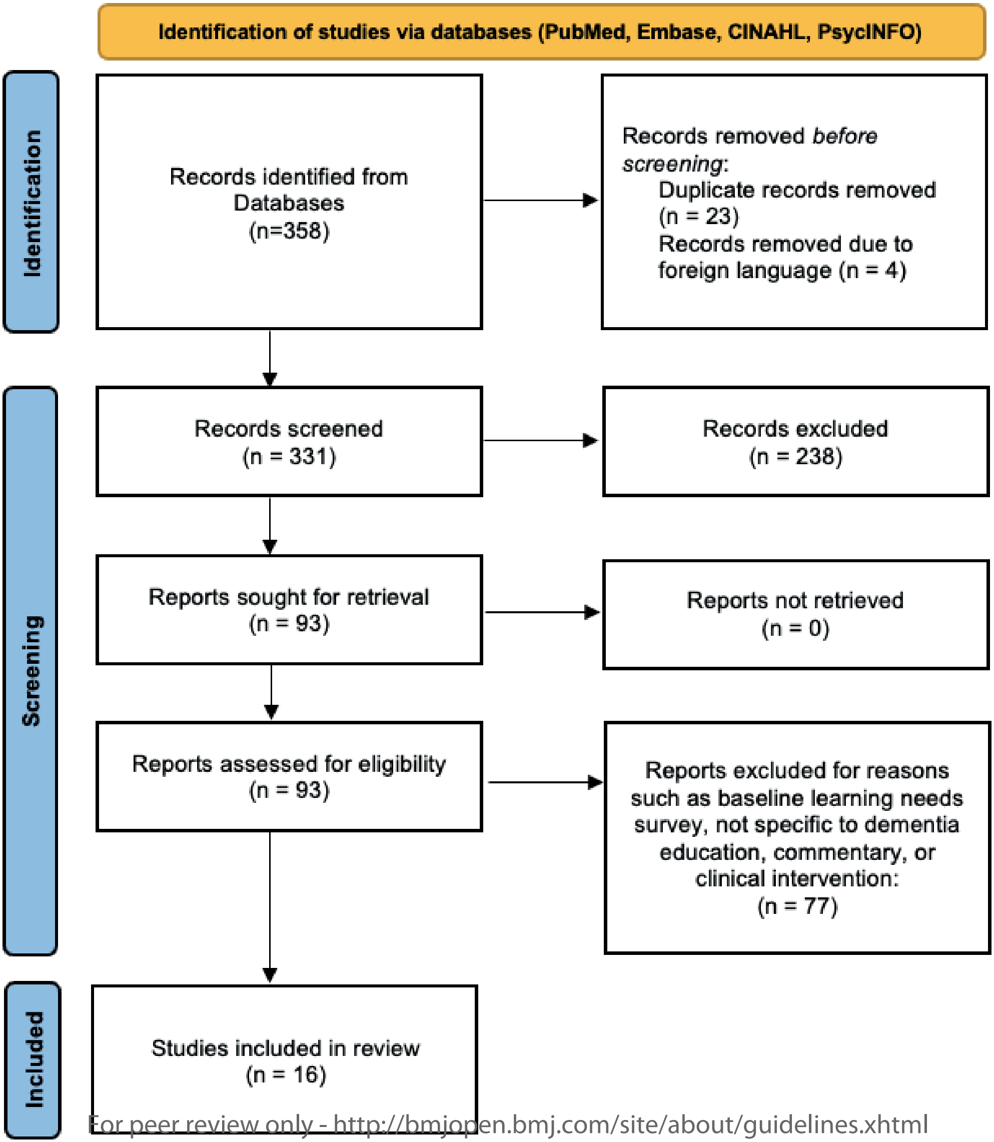

For peer review only - http://bmjopen.bmj.com/site/about/guidelines.xhtml

Figure 2. Initial programme theory

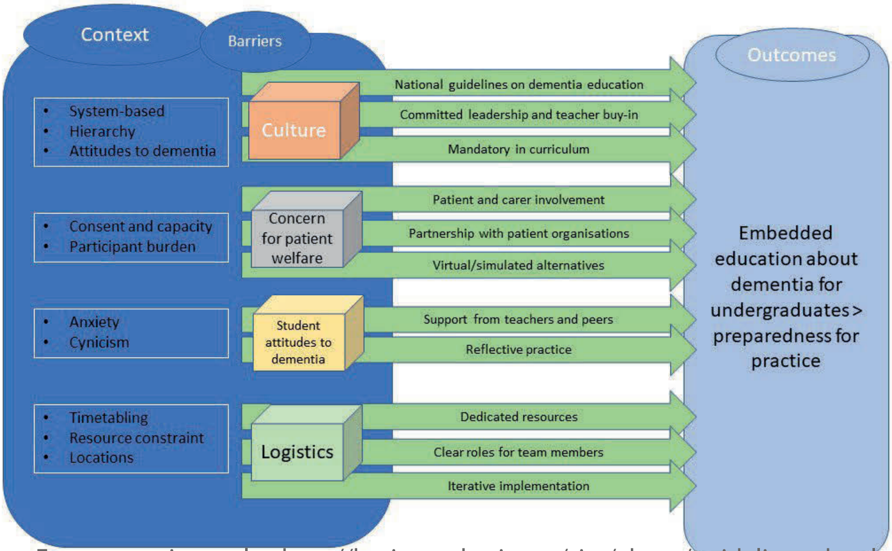

For peer review only - http://bmjopen.bmj.com/site/about/guidelines.xhtml

Appendix 1. Summary of evolution of research project

Initial title: Systematic Review: improving knowledge, skills and attitudes of undergraduate medical students learning to care for older people with cognitive impairment

Modification of **methodology**: Realist review. Which teaching methods are effective in which context?

Modification of **scope:** Exclusion of delirium which has a more acute presentation, in contrast to dementia which requires a longitudinal understanding of the trajectory of the illness and the changing relationship between PWD and their carers

Modification of **title**: How can we support undergraduate medical students to learn to care for older people with dementia? A realist synthesis

Modification of **purpose:** Move away from what is effective to why effective interactive are not widely implemented within curricula

Modification of **title:** What are the barriers and facilitators to integrating effective UG education about dementia into UG medical curricula?

Finalised title: Lost in translation: How can education about dementia be effectively integrated into medical school contexts? A realist synthesis

For peer review only

Supplementary file – full search strategies

PubMed 27/04/21

(((Schools, Medical [MeSH] OR Students, medical [MeSH]) OR Education, medical undergraduate [MeSH]) AND Neurocognitive disorders [MeSH] = 98

Embase 27/04/21[1]

(((Medical student (subject heading) OR undergraduate student (subject heading) OR medical school (subject heading)) AND Cognitive defect (subject heading) = 249

CINAHL 06/10/20[2]

students, medical (CINAHL term) AND delirium, dementia, amnestic, cognitive disorder (CINAHL term) = 0

Psycinfo 27/04/21[3]

(Medical students[4] (subject heading) OR Medical education (subject heading)) AND Cognitive Impairment (subject heading) = 11

No further limitations (eg year of publication or language) applied

---

All databases: 98+249+11 = 358

Eligibility criteria:

Undergraduate or graduate entry medical school programme

Teaching and learning focussing on dementia (rather than delirium or cognitive impairment more broadly)

Evaluating student outcomes > satisfaction, knowledge, skills, attitudes or behaviours

Interventions described clearly enough to classify teaching method

Any research design including quantitative and qualitative

English language

>23 duplicates

>4 foreign language (1 Dutch, 3 German)

>238 excluded by title

= 93 to review in abstract or full text

[1] Search using Ovid Technologies via Newcastle University
[2] Search using CINAHL Information Systems via Newcastle University
[3] Search using American Psychological Association via Newcastle University
[4] Cannot search using term "undergraduate"

For peer review only

Of 93, 77 excluded[5] = 16 eligible papers

For peer review only

[5] Reasons for exclusions could be provided as a itemised list but include: baseline learning needs survey, not specific to dementia education, commentary or clinical intervention.

BMJ Open

Lost in translation: How can education about dementia be effectively integrated into medical school contexts? A realist synthesis

Journal:	*BMJ Open*
Manuscript ID	bmjopen-2023-077028.R2
Article Type:	Original research
Date Submitted by the Author:	11-Oct-2023
Complete List of Authors:	Tullo, Ellen; University of Sunderland, Sunderland Medical School<br>Wakeling, Luisa; Newcastle University, School of Dental Sciences<br>Pearse, Rachel; Northumbria Healthcare NHS Foundation Trust<br>Kheng Khoo, Tien; Griffith University, School of Medicine & Dentistry<br>Teodorczuk, Andrew; The University of Queensland, Faculty of Medicine; The Prince Charles Hospital
**Primary Subject Heading**:	Medical education and training
Secondary Subject Heading:	Geriatric medicine
Keywords:	Dementia, MEDICAL EDUCATION & TRAINING, EDUCATION & TRAINING (see Medical Education & Training)

SCHOLARONE™
Manuscripts

I, the Submitting Author has the right to grant and does grant on behalf of all authors of the Work (as defined in the below author licence), an exclusive licence and/or a non-exclusive licence for contributions from authors who are: i) UK Crown employees; ii) where BMJ has agreed a CC-BY licence shall apply, and/or iii) in accordance with the terms applicable for US Federal Government officers or employees acting as part of their official duties; on a worldwide, perpetual, irrevocable, royalty-free basis to BMJ Publishing Group Ltd ("BMJ") its licensees and where the relevant Journal is co-owned by BMJ to the co-owners of the Journal, to publish the Work in this journal and any other BMJ products and to exploit all rights, as set out in our licence.

The Submitting Author accepts and understands that any supply made under these terms is made by BMJ to the Submitting Author unless you are acting as an employee on behalf of your employer or a postgraduate student of an affiliated institution which is paying any applicable article publishing charge ("APC") for Open Access articles. Where the Submitting Author wishes to make the Work available on an Open Access basis (and intends to pay the relevant APC), the terms of reuse of such Open Access shall be governed by a Creative Commons licence – details of these licences and which Creative Commons licence will apply to this Work are set out in our licence referred to above.

Other than as permitted in any relevant BMJ Author's Self Archiving Policies, I confirm this Work has not been accepted for publication elsewhere, is not being considered for publication elsewhere and does not duplicate material already published. I confirm all authors consent to publication of this Work and authorise the granting of this licence.

For peer review only

Lost in translation: How can education about dementia be effectively integrated into medical school contexts? A realist synthesis

Ellen Tullo

Sunderland Medical School, University of Sunderland, UK

Luisa Wakeling

School of Dental Sciences, Newcastle University, UK

Rachel Pearse

Northumbria NHS Foundation Trust, Newcastle upon Tyne, UK

Tien K Khoo

School of Medicine & Dentistry, Griffith University, Australia; Graduate School of Medicine, University of Wollongong, Australia

Andrew Teodorczuk

Faculty of Medicine, The University of Queensland, Australia; The Prince Charles Hospital, Metro North Mental Health, Australia; School of Medicine and Dentistry, Griffith University, Australia

Correspondence to:

Luisa Wakeling

Faculty of Medical Sciences, Newcastle University, Newcastle upon Tyne, NE2 4HH, UK

luisa.wakeling@ncl.ac.uk

For peer review only

Abstract

Objectives

The prevalence of dementia in both community and hospital settings requires a clinical workforce that is skilled in diagnosis and management of the condition to competently care for patients. Though evidence of successful educational interventions about dementia exist, effective translation into medical school curricula is the exception rather than the norm.

Design

We adopted a realist synthesis approach following RAMESES guidelines to answer the following questions: i) what are the barriers to integrating effective interventions about dementia into medical school curricula, ii) where they are successfully delivered, what are the contextual factors that allow for this enactment?

Data sources

We searched PubMed, Embase, CINAHL, and PsycINFO using the MesH terms *Schools, Medical; Students, Medical; Education, Medical* AND *Neurocognitive disorders,* or the closest possible set of terms within each database.

Eligibility criteria

Undergraduate or graduate entry medical school programme, teaching and learning focussing on dementia, evaluating student outcomes (satisfaction, knowledge, skills, attitudes or behaviours), interventions described clearly enough to classify teaching method, any research design (quantitative and qualitative), English language.

For peer review only - http://bmjopen.bmj.com/site/about/guidelines.xhtml

Data extraction and synthesis

We used a shared spreadsheet to enter key information about eligible studies and the reasons for excluding studies that did not fit eligibility criteria. We extracted descriptive data about the nature of educational interventions and narrative information as to barriers and facilitators to implementing those interventions.

Results

Our initial literature search identified sixteen relevant papers for review. Systematic extraction of data informed the development of an initial programme theory structured around four contextual barriers: "culture", "concern for patient welfare", "student attitudes" and "logistics" with associated facilitatory mechanisms embed medical education about dementia.

Conclusions

We outline the process of generating our initial programme theory, including overlap with Cultural Historical Activity Theory (CHAT). We outline our intention to refine our programme theory through ongoing review of the evidence base and collaboration with stakeholders, with the aim of finalising a model for successful integration of dementia education.

Keywords: cultural historical activity theory, curriculum, dementia, realist synthesis

Strengths and limitations of this study

- Realist reviews allow flexibility to refine study methodology in parallel with literature review in order to explain how complex educational interventions might work (or not) in particular contexts.

For peer review only - http://bmjopen.bmj.com/site/about/guidelines.xhtml

- A realist review deciphers what works for whom, in what circumstances, offering a more pragmatic answer for educators interested in improving medical education about dementia in their own institutions than a traditional systematic review.
- Our study sets out an initial programme theory that can be used as a tool to help educators integrate effective education about dementia into the undergraduate curriculum internationally.
- Our initial programme theory has not yet been reviewed and refined by stakeholders outside of our study team.
- The longitudinal viability of integrating dementia education into the undergraduate curriculum remains unanswered by this study.

Introduction

With the ageing population, dementia is an increasingly prevalent illness. Prevalence increases with age such that one in six people in the UK aged over 80 have dementia. (1) Moreover, there is evidence internationally that care for patients with dementia is poor, and staff feel unequipped to manage people with dementia (PWD). (2, 3) In part this can be attributed to gaps in skills and knowledge, (4) but also attitudes. (5, 6) Attitudes are shaped by early education experiences and doctors as leaders in healthcare have an important role in setting the compass of care. (7)

Unfortunately, education about dementia in medical schools remains patchy. (8) There is piecemeal evidence from international studies that discrete interventions can improve students' knowledge of and attitudes towards PWD. Even brief interventions that incorporate elements of experiential learning such as simulation; (9-11) and storytelling (12) have been shown to have at least a short-term positive impact on student knowledge and attitudes, although longer-term impacts and sustainability remain unclear. This may be because of the

For peer review only - http://bmjopen.bmj.com/site/about/guidelines.xhtml

fact that health care settings are not set up to facilitate good dementia practice. Care home placements and clinical clerkships facilitating contact with PWD likewise appear to improve knowledge and attitudes, (13, 14) but are infrequently implemented.

There are also examples of more extensive educational interventions in the form of longitudinal pairings of medical students with PWD. In the United States (US) the PAIRS Program, (15) developed from an original Buddy Program (16) brings together medical students and PWD over a period of one year. Mixed methods evaluation suggests enhanced knowledge, skills and positive attitudes amongst medical students who participated, although students were self-selected and therefore likely to have held predisposing positive attitudes.

The PAIRS model has been adapted in the form of the two-year *Time for Dementia* programme, run collaboratively between a medical school and the UK Alzheimer's Society. (17) Evaluation of *Time for Dementia* is ongoing, but there is evidence from a controlled trial that the intervention led to improvements in medical students' knowledge and attitudes, (18) and also has potential benefits for the patients and families. (19)

Longitudinal programmes involving PWD however are the exception rather than the norm – medical school education about dementia remains inadequate to sufficiently equip graduates to meet the needs of PWD. Given the imperative to improve care for PWD and the existence of evidence as to effective practice, it remains unclear why educational interventions about dementia are not implemented more widely across medical schools. Arguably, this is because curricula are predicated on rigid historically structured content; to bring in change would involve challenging the traditional status quo as "ologies" risk being replaced and thereby devalued. Moreover, systems of care may be slow to adopt good dementia practice and so the hidden curriculum limits the ability to innovate and perceived importance of good dementia education initiatives.

For peer review only - http://bmjopen.bmj.com/site/about/guidelines.xhtml

Rationale and research intent

Our initial intention was to review existing educational interventions about dementia to identify which features of such interventions led to enhancement in medical students' knowledge, skills, attitudes and behaviours. However, our initial review of the literature, including studies cited above, suggested sufficient evidence of a range of effective models of teaching and learning about dementia. Aligned to the research intent the more pertinent questions are, firstly, why effective interventions are not more frequently and systematically integrated into medical school curricula? Secondly, for whom and in which circumstances is successful dementia education effectively implemented in medical school curricula? We reasoned that this question might be better answered using a realist synthesis rather than a more traditional systematic review. (20) Such an approach aims to decipher what works for whom, in what circumstance and is therefore likely to provide a more helpful answer for educators interested in improving medical education about dementia in their own institutions.

Methods

Changes to the review process

As outlined above, the focus and purpose of our review has already evolved. An accepted feature of realist synthesis is that purpose, scope, inclusion criteria and method of data synthesis are refined in an iterative manner as the review progresses. (21) Details of the evolution of our study purpose is included as Appendix 1. Our report is structured according to RAMESES publication standards. (22)

Rationale for realist approach

We have suggested that there is already sufficient evidence as to effective methods for delivering medical education about dementia and an implementation gap. Therefore, a traditional systematic review to highlight key features of effective practice is unlikely to

For peer review only - http://bmjopen.bmj.com/site/about/guidelines.xhtml

answer the question as to why education about dementia remains under-represented in medical curricula or align to the research intent. Rather, there is a need to improve understanding of the barriers to implementation and the facilitators in institutions that have successfully introduced effective practice.

Realist reviews add explanation as to how interventions might work (or not) in particular contexts to produce change. (23) The determinant of a desired outcome, in this case improving medical students' ability to care for PWD, are unlikely to be simple or linear – rather, multiple mechanisms are likely to be relevant. (21) These underlying mechanisms may not be intrinsic to the educational intervention itself, but dependent on the historical context, location and the participants involved. For example, in the case of dementia, public and professional attitudes (sometimes ageist) have changed significantly over the last decades, (5) and this may have a bearing on the gap between the expanding needs of PWD and its proportional under-representation in the medical curriculum. A traditional systematic review is unlikely to capture this contextual issue.

A realist approach has been used to examine the complex underpinnings of a range of polemics in medical education including supporting international medical graduates into the UK workforce, (24) the supervision and appraisal process of doctors (25, 26), and how healthcare training translates into benefits for patients. (27) A realist synthesis will similarly allow us to develop a "programme theory" – a diagram to lay out the contexts, mechanisms and outcomes in relation to the curricular integration of UG education about dementia.

Data sources

We drafted the following eligibility criteria before conducting an exploratory search of databases in (PubMed, Embase, CINAHL, PsycINFO) using the MesH terms *Schools, Medical; Students, Medical; Education, Medical* AND *Neurocognitive disorders,* or the

For peer review only - http://bmjopen.bmj.com/site/about/guidelines.xhtml

closest possible set of terms within each database (see Supplementary file for details of the literature searches).

Eligibility criteria

- Undergraduate or graduate entry medical school programme
- Teaching and learning focussing on dementia (rather than delirium or cognitive impairment more broadly)
- Evaluating student outcomes > satisfaction, knowledge, skills, attitudes or behaviours
- Interventions described clearly enough to classify teaching method
- Any research design including quantitative and qualitative
- English language

Data extraction and synthesis

We divided the articles between our research team and constructed a shared spreadsheet to enter key information about eligible studies and to keep track of the reason for excluding studies that did not fit eligibility criteria. We extracted descriptive data about the nature of educational interventions and narrative information as to barriers and facilitators to implementing those interventions. We used the template as a basis for discussion of key findings at regular team meetings and to note any concerns about quality of the included studies.

We derived our IPT from synthesis of the key barriers and facilitators to implementation emerging from the initial literature review and data extraction as described in the results. This is the first step in our process of realist synthesis – proposed ongoing realist methodology to iteratively test and refine the model is outlined in the discussion.

For peer review only - http://bmjopen.bmj.com/site/about/guidelines.xhtml

Patient and public involvement

None at this stage.

Results

Literature search

Our initial database searches yielded 358 papers. Twenty-three were duplicates, 238 were excluded by title and four were not in English. We reviewed 93 papers by abstract or full text. Seventy-seven papers did not meet our eligibility criteria, leaving sixteen studies for data extraction (Figure 1).

Manual searching of the reference lists of the sixteen eligible studies, and our team's awareness of pertinent literature beyond the remit of the search highlighted that our initial database search terms had not been sufficiently broad to identify all relevant interventions. This may be due to medical education research being referenced across different database types (biomedical, psychiatric, educational) that each use a range of search terms in relation to dementia, medical education and the pre-qualification stage of medical training. We included a further four relevant studies in our initial synthesis to inform our IPT (Table 1).

Document characteristics

Of the twenty papers evaluating UG dementia education, thirteen involved only medical students and five involved other healthcare professions including pharmacy, physician assistants and nurses. One study involved staff contributing to an educational programme. Cohort sizes ranged from twelve to 386 and the lengths of the educational interventions ranged from one-hour to two-years. The twenty studies considered a range of outcomes including student knowledge (n=7), student attitudes (n=9) and qualitative data collected from focus groups, interviews and student reflections (n=9).

For peer review only - http://bmjopen.bmj.com/site/about/guidelines.xhtml

Initial findings

Extraction of narrative data about barriers to and facilitators of implementation of each intervention revealed a number of common themes. Many studies initially highlighted the barriers towards implementing prior educational interventions about dementia as a rationale for the development of their own.

The evolution of medical education is influenced by the historical and cultural context in which it takes place. There has been a strong reliance on Flexner models of curricula that are over 100 years old - it is surprising how slowly curricular structures and content has evolved. (28) An emphasis on system-based specialties and academic disciplines means that conditions such as dementia do not

'belong' to a particular specialty and therefore may not have systematically appeared in the UG medical curriculum. This has been highlighted by national guidelines in the UK and elsewhere, leading to mandated dementia education, cited by some authors as a prompt for their institution to introduce this. Despite such a mandate, authors note persistent negative attitudes towards dementia amongst faculty which act as a barrier to implementation. The presence of a committed leader and faculty engagement appears to be an important facilitator to integration of dementia education.

Authors cited understandable concerns about the welfare of PWD who might have limited capacity to consent to participation in teaching, and on the potential burden to PWD and their carers. Teams that worked in partnership with PWD, carers, and voluntary sector organisations in the design and delivery of dementia education were able to navigate some of these risks. Other studies elected to use virtual or simulated alternatives rather than direct contact with PWD and their carers.

The attitudes of medical students were also of relevance – there were examples of negative attitudes towards dementia as a topic, with student perception that community-based

For peer review only - http://bmjopen.bmj.com/site/about/guidelines.xhtml

teaching about dementia detracted from the more pertinent environment of acute care. As such, students were less motivated to learn about chronic conditions such as dementia in community placements than they were about acute presentations of illnesses in emergency environments. This may be associated with institutional culture that devalues care of older adults, with the negative attitudes of faculty influencing medical students, sometimes termed the 'hidden curriculum'. Despite negative attitudes, successful programmes of education about dementia instilled motivation in students through mechanisms such as sustained supervision from teachers and peers, and through systematic reflective practice.

Authors also cited the substantial logistical challenges to integration of dementia education, most notably longitudinal programmes pairing medical students with PWD. A full timetable and resource constraints challenged curriculum designers, whereas dedicated resources, administrative support, clear roles for faculty members and iterative implementation acted as facilitators to integrate dementia education.

One paper specifically explored the barriers and facilitators to implementing a longitudinal dementia education programme in a single site. (29) Their findings overlap substantially with the themes appearing across other studies, affording some validity to our initial explanation of contexts, mechanisms and outcomes in the following IPT.

Initial programme theory (IPT)

An IPT (also known as draft or rough programme theory) *'refers to the initial sketch of a theory that is used to guide a realist synthesis'*. (21) We synthesised the findings from our exploratory literature search with our own knowledge and experience of designing and delivering medical education about dementia to produce the following IPT illustrating the contexts where teaching and learning takes place, the mechanisms (barriers and facilitators to integration into the curriculum) and the desired outcomes, often termed CMOs (Figure 2).

For peer review only

We condensed and structured our initial findings into four contextual barriers to implementation of effective medical education: culture, concern for patient welfare, student attitudes to dementia, and logistics. 'Culture' concerns the rigidity of curricular structures and the perceived lower status of conditions such as dementia in the hierarchy of 'ologies'. 'Concern for patient welfare' incorporates legitimate concerns about the potential vulnerability of PWD and their carers to be burdened by involvement in education. 'Student attitudes' acknowledges the perception that clinical experience of acute care is a more valuable learning experience than community-based engagement with PWD. 'Logistics' highlight the resource implications in terms of time and money to implement longitudinal interventions for large numbers of students. Each of the four contextual barriers are accompanied by mechanisms that facilitate surmounting these barriers in order to achieve the outcome of embedded education which prepares medical students for graduation to practice.

Discussion

Our IPT proposes four categories of barriers to implementation of effective dementia education which derive from the medical school context: culture, concern for patient welfare, student attitudes and logistics. These contextual barriers might be overcome by a range of mechanisms in order to lead to the desired outcome of embedded education that adequately prepares graduates to care for PWD. Realist synthesis encourages the development of programme theory through comparison with existing 'middle range theories' – theories available in the wider literature that are relevant to the topic but may come from close or peripheral academic disciplines.

In this case we found that Cultural Historical Activity Theory (CHAT) held important overlap and relevance with our IPT. CHAT, or social activity theory, is of particular relevance as it is a theory that targets learning at an organisational or systems level and can be applied to help

For peer review only - http://bmjopen.bmj.com/site/about/guidelines.xhtml

bring about educational change. Specifically, it is also relevant in circumstances where multiple voices are present. The fact that the current medical school curricula, and approaches to change, are bound by historical and deeply engrained cultural practice further highlights the relevance as typically it allows challenging of the status quo. It has been applied to dementia settings previously where innovative approaches to systems learning around dementia care have been applied. For example, in the paper by Teodorczuk et al (2015), by defining the object as to learn about the person, its application let to innovative solutions such as relaxing visiting hours to further learn and align with the purpose of the system. (30) . Moreover, multiple voices will allow engagement with the practice context and degree of dementia capability that may invariably limit implementation of any education innovations.

At play are multiple tensions that block embedding of newer dementia-focussed approaches. According to CHAT these can be points of further expansive learning and by this manner a more fit for purpose curriculum can be developed. (31) Current divisions of labour both within education and clinical practice can be identified, and suggestions made to overcome logistical challenges to embedding more dementia focussed curricula. Arguably such an approach will also lead to medical education practice becoming more patient-focussed when some have critiqued the absence of the patient in medical education. (32)

A key strength of this realist synthesis has been the flexibility to refine our study methodology in order to explain how complex educational interventions might work (or not) in particular contexts. We believe that this approach will lead to a more pragmatic answer for educators interested in improving medical education about dementia in their own institutions than a traditional systematic review. At this stage our initial programme theory has not yet been reviewed and refined by stakeholders outside of our study team – this limitation will be addressed in the next stage of our project. We will also address the longitudinal viability of

For peer review only - http://bmjopen.bmj.com/site/about/guidelines.xhtml

integrating dementia education into the undergraduate curriculum which remains unanswered at this stage.

We propose to use our IPT as the basis for ongoing realist synthesis – we will further interrogate the literature around medical education on dementia to evaluate the fit of our model and make refinements as required. We will test the refined programme theory using case-studies of successful and unsuccessful implementation of educational interventions into medical school curricula, including neglected areas of teaching that may face similar factors such as learning disability. We will seek external validation of the programme theory through consultation with educators with experience of successfully integrating educational interventions about dementia into medical curricula, in addition to people with dementia and their carers. This will also aid our understanding of the longitudinal viability of integrating dementia education – published articles usually outline the immediate outcomes of an intervention, but seldom contain information about the longer-term impact of curriculum reform. Discussions with medical educators who have spent years in the field will help to shed light on a process that can be slow to evolve.

Conclusion

Our IPT provides a firm basis for moving forward with our understanding of effective implementation of dementia teaching into medical education. We acknowledge that the integration of medical education about dementia is likely to be challenging in the context of established medical culture, but the changing demographics of our population and the needs of PWD warrants social accountability to do so. Realist methodology, including the application of systems change models such as CHAT, will help us to better understand the way that contextual barriers to effective medical education about dementia can be overcome.

For peer review only - http://bmjopen.bmj.com/site/about/guidelines.xhtml

Contributors: ET conceptualised the idea, approach, and constructed the research team. ET, LW, RP, TK and AT provided feedback and further developed the idea and realist synthesis approach. RP was responsible for the primary search of initial studies. ET, LW, RP, TK and AT contributed to the search refinement, data extraction and analysis. ET summarised findings and drafted an initial programme theory to which LW, RP, TK and AT provided feedback and further developed. ET prepared the initial draft manuscript and LW, RP, TK and AT reviewed and developed the manuscript. All authors (ET, LW, RP, TK and AT) accepted the final version.

Acknowledgments: With thanks to Millie Kehoe for her advice on the realist approach and structuring our report.

Competing interests: The authors report no competing interests.

Funding: The authors report no funding associated with this work.

Data availability statement: No new data were created during this study.

Ethics approval: Not required for literature review.

Open Access: This is an open access article distributed in accordance with the Creative Commons Attribution Non Commercial (CC BY-NC 4.0) license, which permits others to distribute, remix, adapt, build upon this work non-commercially, and license their derivative works on different terms, provided the original work is properly cited, appropriate credit is given, any changes made indicated, and the use is non-commercial. See: http://creativecommons.org/licenses/by-nc/4.0/.

For peer review only

Table 1. Papers included in exploratory literature review

**Author**	**Year**	**Title**	**Participants**	**Outcome measure**
Annear et al (13)	2016	Encountering aged care: a mixed methods investigation of medical students' clinical placement experiences	61 Y5 MS	Knowledge (DKAT)<br>Qualitative (FG)
**Banerjee et al (17)**	2017	How do we enhance undergraduate healthcare education in dementia?	n/a	n/a
**Banerjee et al (18)**	2021	A comparative study of the effect of the Time for Dementia programme on medical students	386 Y2/3 MS	Knowledge (ADKS, DKQ)<br>Attitudes (ADQ, DAS, MCRS, JSE)
Blazek et al (33)	2015	Medical Students in a Dementia Care Facility: and Enhanced Geriatric Psychiatry Experience	99 Y3 MS	Knowledge (Likert)<br>Attitudes (Likert)<br>Qualitative (FG)
de Abreu et al (9)	2017	A Simulation Exercise to Raise Learners' Awareness of the Physical and Cognitive Changes in Older Adults	49 students (MS, physician assistant, pharmacy, psychiatry residents)	Attitudes (ADQ)
**Feeney at al (29)**	2021	Barriers and facilitators to implementing a longitudinal dementia education programme into undergraduate healthcare curricula: a qualitative study	12 staff	Qualitative (Interviews)
George et al (34)	2011	Impact of participation in TimeSlips, a creative group-based storytelling program, on medical student attitudes toward persons with dementia: a qualitative study.	15 Y4 MS	Qualitative (Questionnaires)
George et al (35)	2012	Evaluating an arts-based intervention to improve medical student attitudes toward persons with dementia using the dementia attitudes scale.	22 Y4 MS	Attitudes (DAS)
George et al (36)	2013	An arts-based intervention at a nursing home to improve medical students' attitudes toward persons with dementia	22 Y4 MS	Attitudes (DAS)
George et al (12)	2014	How a creative storytelling intervention can improve medical student attitude towards persons with dementia: a mixed methods study	22 Y4 MS	Attitudes (DAS)<br>Qualitative (FG)
Gilmartin-Thomas et al (37)	2018	Impact of a Virtual Dementia Experience on Medical and Pharmacy Students' Knowledge and Attitudes Toward People with Dementia: A Controlled Study	278 Y3MS and y4 pharmacy students	Attitudes (DAS)

For peer review only - http://bmjopen.bmj.com/site/about/guidelines.xhtml

**Gilmartin-Thomas et al (10)**	2020	Qualitative evaluation of how a virtual dementia experience impacts medical and pharmacy students' self-reported knowledge and attitudes towards people with dementia	53 Y3 MS and Y4 pharmacy students	Qualitative (FG)
Goldman et al (38)	2019	A qualitative study of the impact of a dementia experiential learning project on pre-medical students: a friend for Rachel	95 pre-MS	Qualitative (reflections)
Griffiths et al (39)	2020	Perceptions and attitudes towards dementia among university students in Malaysia	97 MS and pharmacy students (Y unknown)	Attitudes (AADS, ATOP, IPQ)
Jefferson et al (15)	2012	Medical student education program in Alzheimer's disease: the PAIRS Program.	45 Y1 MS	Knowledge (BPDKT, BUPPDKT)<br>Qualitative (reflections)
Matsumura et al (11)	2018	Simulating Clinical Psychiatry for Medical Students: A Comprehensive Clinic Simulator with Virtual Patients and an Electronic Medical Record System	79 Y5 MS	Knowledge (non-validated)
McCaffrey et al (40)	2013	Interprofessional education in community-based Alzheimer's disease diagnosis and treatment	110 MS and nurse practitioner trainees	Knowledge (non-validated)<br>Attitudes (non-validated)
Morhardt (16)	2013	The effects of an experiential learning and mentorship program pairing medical students and persons with cognitive impairment: A qualitative content analysis.	71 Y1 MS	Qualitative (reflections)
Van Zuilen et al (14)	2018	Achieving medical student mastery in screening for cognitive impairment: Results from a blended learning curriculum	60 MS, (Y unknown)	Knowledge (non-validated)
Yang et al (41)	2016	3D (dementia, delirium, depression) didactic for medical students.	23 Y3/4 MS	Knowledge (non-validated)

Papers identified through manual searches in **bold**.
AADS – Adolescent Attitude to Dementia Scale.
ADKS – Alzheimer's Disease Knowledge Scale.
ADQ – Approaches to Dementia Questionnaire.
ATOP – Attitudes Towards Older People.
BPDKT – Buddy Programme Dementia Knowledge Test.
BUPPDKT – Boston University Pairs Programme Knowledge Test.
DAS – Dementia Attitudes Scale.
DKAT – Dementia Knowledge Assessment Survey.
DKQ – Dementia Knowledge Questionnaire.
FG – Focus Groups.

For peer review only - http://bmjopen.bmj.com/site/about/guidelines.xhtml

IPQ – Illness Perception Questionnaire.
JSE – Jefferson Scale of Empathy.
MCRS – Medical Condition Regard Scale.
MS – medical student.
Y – year.

For peer review only

For peer review only - http://bmjopen.bmj.com/site/about/guidelines.xhtml

References

1. Alzheimer's Research UK. *Prevalence.* 2022 Available from: https://www.dementiastatistics.org/statistics-about-dementia/prevalence-2/. [Accessed Sep 2022]

2. Alzheimer's Society. *From diagnosis to end of life: the lived experiences of dementia care and support.* 2020. Available from: https://www.alzheimers.org.uk/sites/default/files/2020-10/pathway_report_full_final.pdf. [Accessed May 2022]

3. [ADI] Alzheimer's Disease International. *World alzheimer report 2021: journey through the diagnosis of dementia*. 2021. Available from: https://www.alzint.org/u/World-Alzheimer-Report-2021.pdf. [Accessed May 2022]

4. Tullo ES, Gordon AL. Teaching and learning about dementia in UK medical schools: a national survey. *BMC Geriatrics*. 2013;13(1):29.

5. [ADI] Alzheimer's Disease International. *World alzheimer report 2019: attitudes to dementia.* 2019 Available from: https://www.alzint.org/u/WorldAlzheimerReport2019.pdf. [Accessed May 2022]

6. Royal College of Psychiatrists. *Who cares wins. Improving the outcome for older people admitted to the general hospital: guidelines for the development of liaison mental health services for older people.* London: Royal College of Psychiatrists (RCPsych); 2005.

7. Martin J, Dacre J. Professional attitudes: why we should care. *Clin Med (Lond).* 2002;2(3):182-4.

8. Tullo E, Allan L. What should we be teaching medical students about dementia? *Int Psychogeriatr*. 2011;23(7):1044-50.

9. de Abreu ID, Hinojosa-Lindsey M, Asghar-Ali AA. A Simulation Exercise to Raise Learners' Awareness of the Physical and Cognitive Changes in Older Adults. *Acad Psychiatry*. 2017;41(5):684-7.

10. Gilmartin-Thomas JF, McNeil J, Powell A, Malone DT, Larson IC, O'Reilly CL, et al. Qualitative evaluation of how a virtual dementia experience impacts medical and pharmacy students' self-reported knowledge and attitudes towards people with dementia. *Dementia (London).* 2020;19(2):205-20.

11. Matsumura Y, Shinno H, Mori T, Nakamura Y. Simulating Clinical Psychiatry for Medical Students: a Comprehensive Clinic Simulator with Virtual Patients and an Electronic Medical Record System. *Acad Psychiatry.* 2018;42(5):613-21.

12. George DR, Stuckey HL, Whitehead MM. How a creative storytelling intervention can improve medical student attitude towards persons with dementia: a mixed methods study. *Dementia (London)*. 2014;13(3):318-29.

For peer review only - http://bmjopen.bmj.com/site/about/guidelines.xhtml

13. Annear MJ, Lea E, Lo A, Tierney L, Robinson A. Encountering aged care: a mixed methods investigation of medical students' clinical placement experiences. *BMC Geriatrics.* 2016;16(1):38.

14. Van Zuilen R, Cabrera K, Danisi J, Palacios J. Achieving medical student mastery in screening for cognitive impairment: results from a blended learning curriculum. *JAGS*. 2018;66 S81-S2.

15. Jefferson AL, Cantwell NG, Byerly LK, Morhardt D. Medical student education program in Alzheimer's disease: The PAIRS Program. *BMC Medical Education*. 2012;12(1):80.

16. Morhardt D. The effects of an experiential learning and mentorship program pairing medical students and persons with cognitive impairment: a qualitative content analysis. [dissertation]. Chicago: Loyola University; 2013.

17. Banerjee S, Farina N, Daley S, Grosvenor W, Hughes L, Hebditch M, et al. How do we enhance undergraduate healthcare education in dementia? A review of the role of innovative approaches and development of the Time for Dementia Programme. *Int J Geriatr Psychiatry.* 2017;32(1):68-75.

18. Banerjee S, Jones C, Wright J, Grosvenor W, Hebditch M, Hughes L, et al. A comparative study of the effect of the Time for Dementia programme on medical students. *Int J Geriatr Psychiatry*. 2021;36(7):1011-9.

19. Cashin Z, Daley S, Hebditch M, Hughes L, Banerjee S. Involving people with dementia and their carers in dementia education for undergraduate healthcare professionals: a qualitative study of motivation to participate and experience. *Int Psychogeriatr*. 2019;31(6):869-76.

20. Pawson R, Greenhalgh T, Harvey G, Walshe K. Realist review--a new method of systematic review designed for complex policy interventions. *J Health Serv Res Policy*. 2005;10 Suppl 1:21-34.

21. Wong G, Westhorp G, Pawson R, Greenhlagh T. Realist Synthesis. RAMESES Training Materials. 2013. Available from: https://www.ramesesproject.org/media/Realist_reviews_training_materials.pdf. [Accessed July 2022]

22. Wong G, Greenhalgh T, Westhorp G, Buckingham J, Pawson R. RAMESES publication standards: realist syntheses. *BMC Medicine*. 2013;11(1):21.

23. Pawson R, Tilley N. *Realistic Evaluation*. London: Sage; 1997.

24. Kehoe A, McLachlan J, Metcalf J, Forrest S, Carter M, Illing J. Supporting international medical graduates' transition to their host-country: realist synthesis. *Med Educ*. 2016;50(10):1015-32.

25. Brennan N, Bryce M, Pearson M, Wong G, Cooper C, Archer J. Understanding how appraisal of doctors produces its effects: a realist review protocol. *BMJ Open*. 2014;4:e005466.

For peer review only - http://bmjopen.bmj.com/site/about/guidelines.xhtml

26. Rees CE, Lee SL, Huang E, Denniston C, Edouard V, Pope K, et al. Supervision training in healthcare: a realist synthesis. *Adv Health Sci Educ Theory Pract.* 2020;25(3):523-61.

27. Illing J, Corbett S, Kehoe A, Carter M, Hesselgreaves H, Crampton P, et al. *How Does the Education and Training of Health and Social Care Staff Transfer to Practice and Benefit Patients? A Realist Approach*. Department of Health; 2019.

28. Jason H, Douglas A. Are the conditions right for a 21st-century medical school? *Lancet*. 2015;385(9969):672-3.

29. Feeney Y, Daley S, Flaherty B, Banerjee S. Barriers and facilitators to implementing a longitudinal dementia education programme into undergraduate healthcare curricula: a qualitative study. *BMC Medical Education*. 2021;21(1):201.

30. Teodorczuk A, Mukaetova-Ladinska E, Corbett S, Welfare M. Deconstructing dementia and delirium hospital practice: using cultural historical activity theory to inform education approaches. *Adv Health Sci Educ Theory Pract*. 2015;20(3):745-64.

31. Teodorczuk A, Morris C. Time to CHAT about globalisation. *Med Educ*. 2019;53(1):3-5.

32. Teodorczuk A, Yardley S, Patel R, Rogers GD, Billett S, Worley P, et al. Medical education research should extend further into clinical practice. *Med Educ*. 2017;51(11):1098-100.

33. Blazek MC, Struble L, Cavalcante A, Masse J. Medical Students in a Dementia Care Facility: an Enhanced Geriatric Psychiatry Experience. *Acad Psychiatry.* 2016;40(6):932-4.

34. George DR, Stuckey HL, Dillon CF, Whitehead MM. Impact of participation in TimeSlips, a creative group-based storytelling program, on medical student attitudes toward persons with dementia: a qualitative study. *Gerontologist*. 2011;51(5):699-703.

35. George DR, Yang C, Stuckey HL, Whitehead MM. Evaluating an arts-based intervention to improve medical student attitudes toward persons with dementia using the dementia attitudes scale. *J Am Geriatr Soc*. 2012;60(8):1583-5.

36 George DR, Stuckey HL, Whitehead MM. An arts-based intervention at a nursing home to improve medical students' attitudes toward persons with dementia. *Acad Med*. 2013;88(6):837-42.

37 Gilmartin-Thomas JF, McNeil J, Powell A, Malone DT, Wolfe R, Larson IC, et al. Impact of a Virtual Dementia Experience on Medical and Pharmacy Students' Knowledge and Attitudes Toward People with Dementia: A Controlled Study. *J Alzheimers Dis*. 2018;62(2):867-76.

For peer review only - http://bmjopen.bmj.com/site/about/guidelines.xhtml

38 Goldman JS, Trommer AE. A qualitative study of the impact of a dementia experiential learning project on pre-medical students: a friend for Rachel. *BMC Medical Education*. 2019;19(1):127.

39 Griffiths AW, Cheong WL, Saw PS, Parveen S. Perceptions and attitudes towards dementia among university students in Malaysia. *BMC Medical Education*. 2020;20(1):82.

40. McCaffrey R, Tappen RM, Lichtstein DM, Friedland M. Interprofessional education in community-based Alzheimer's disease diagnosis and treatment. *J Interprof Care*. 2013;27(6):534-6.

41. Yang M, Graham A, Cruz T, Gamaldo C, Salas R, Lehmann S, et al. 3D (dementia, delirium, depression) didactic for medical students *JAGS*. 2016;64:S178.

42. Page MJ, McKenzie JE, Bossuyt PM, Boutron I, Hoffmann TC, Mulrow CD, et al. The PRISMA 2020 statement: an updated guideline for reporting systematic reviews. *BMJ*. 2021;327:n71

For peer review only

FIGURE TITLES

Figure 1. PRISMA flow diagram for study selection process (42)

Figure 2. Initial programme theory

For peer review only - http://bmjopen.bmj.com/site/about/guidelines.xhtml

Figure 1. PRISMA 2020 flow diagram for study selection process (42)

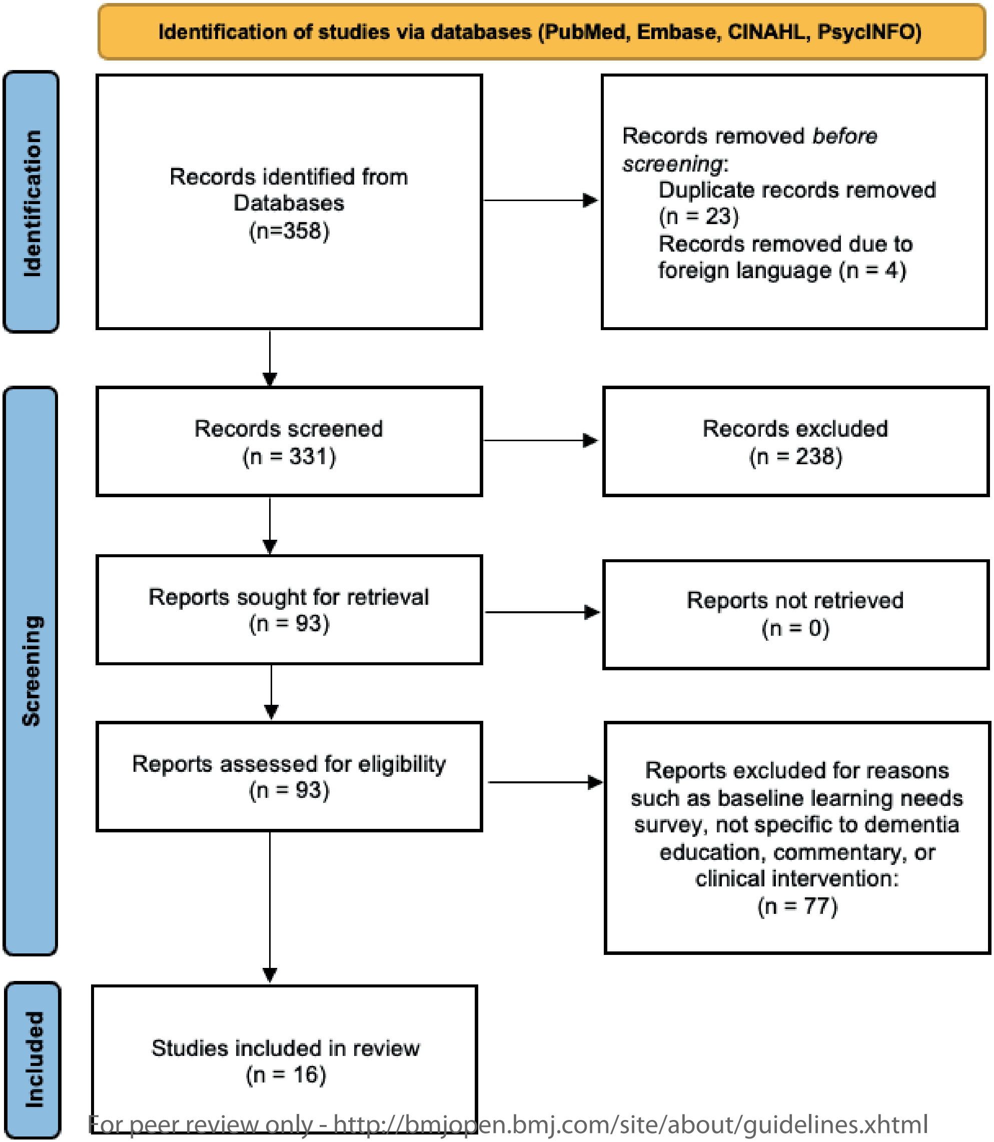

For peer review only - http://bmjopen.bmj.com/site/about/guidelines.xhtml

Figure 2. Initial programme theory

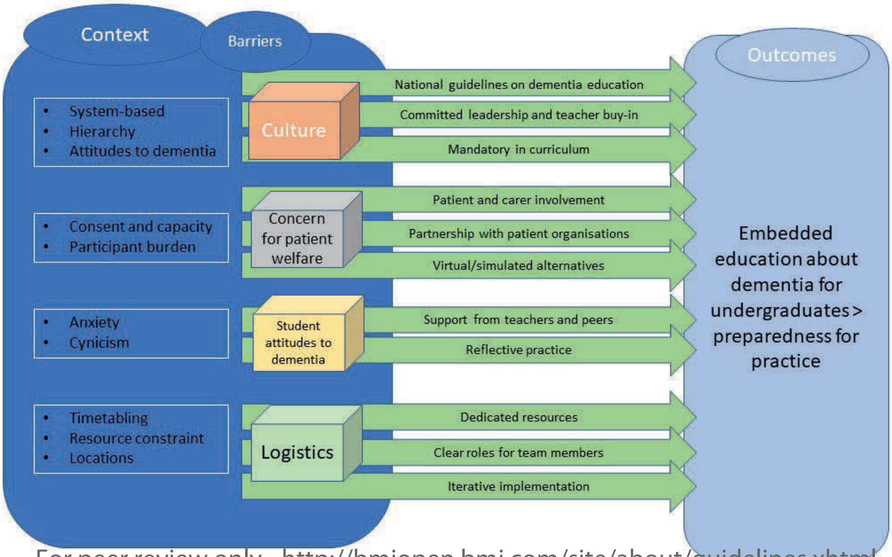

For peer review only - http://bmjopen.bmj.com/site/about/guidelines.xhtml

Appendix 1. Summary of evolution of research project

Initial title: Systematic Review: improving knowledge, skills and attitudes of undergraduate medical students learning to care for older people with cognitive impairment

Modification of **methodology**: Realist review. Which teaching methods are effective in which context?

Modification of **scope:** Exclusion of delirium which has a more acute presentation, in contrast to dementia which requires a longitudinal understanding of the trajectory of the illness and the changing relationship between PWD and their carers

Modification of **title**: How can we support undergraduate medical students to learn to care for older people with dementia? A realist synthesis

Modification of **purpose:** Move away from what is effective to why effective interactive are not widely implemented within curricula

Modification of **title:** What are the barriers and facilitators to integrating effective UG education about dementia into UG medical curricula?

Finalised title: Lost in translation: How can education about dementia be effectively integrated into medical school contexts? A realist synthesis

For peer review only

Supplementary file – full search strategies

PubMed 27/04/21

(((Schools, Medical [MeSH] OR Students, medical [MeSH]) OR Education, medical undergraduate [MeSH]) AND Neurocognitive disorders [MeSH] = 98

Embase 27/04/21[1]

(((Medical student (subject heading) OR undergraduate student (subject heading) OR medical school (subject heading)) AND Cognitive defect (subject heading) = 249

CINAHL 06/10/20[2]

students, medical (CINAHL term) AND delirium, dementia, amnestic, cognitive disorder (CINAHL term) = 0

Psycinfo 27/04/21[3]

(Medical students[4] (subject heading) OR Medical education (subject heading)) AND Cognitive Impairment (subject heading) = 11

No further limitations (eg year of publication or language) applied

---

All databases: 98+249+11 = 358

Eligibility criteria:

Undergraduate or graduate entry medical school programme

Teaching and learning focussing on dementia (rather than delirium or cognitive impairment more broadly)

Evaluating student outcomes > satisfaction, knowledge, skills, attitudes or behaviours

Interventions described clearly enough to classify teaching method

Any research design including quantitative and qualitative

English language

>23 duplicates

>4 foreign language (1 Dutch, 3 German)

>238 excluded by title

= 93 to review in abstract or full text

[1] Search using Ovid Technologies via Newcastle University
[2] Search using CINAHL Information Systems via Newcastle University
[3] Search using American Psychological Association via Newcastle University
[4] Cannot search using term "undergraduate"

For peer review only

Of 93, 77 excluded[5] = 16 eligible papers

[5] Reasons for exclusions could be provided as a itemised list but include: baseline learning needs survey, not specific to dementia education, commentary or clinical intervention.

For peer review only
